# Screening and mutagenesis breeding of *B. A salt* YB13 for high producing tetramethylpyrazine and the application of its fortified *Daqu* for improving the quality of *mixed-flavor baijiu*

**DOI:** 10.1016/j.fochx.2025.103401

**Published:** 2025-12-11

**Authors:** Qing Zhao, Di Wu, Biao Hao, Jie Zhang, Tianquan Pan, Jie Qiao, Huawei Zeng, Hongwen Yang

**Affiliations:** a Anhui Province Key Laboratory of Pollutant Sensitive Materials and Environmental Remediation, Department of Bioengineering, College of Life Science, Huaibei Normal University, Huaibei 235000, Anhui, China; b Technology Center of Enterprise, Anhui Jinzhongzi Distillery Co., Ltd., Fuyang 236023, Anhui, China

**Keywords:** *B. Halotolerans*, High producing tetramethylpyrazine, Fortified Daqu, Microbial community, Flavor compounds

## Abstract

This study isolated *B. halotolerans* YB13 from high-temperature Daqu and enhanced its tetramethylpyrazine (TTMP) yield to 2.98 g/kg through screening and plasma mutagenesis. The fortified Daqu containing YB13 was used in Mixed-flavor Baijiu fermentation. Compared to the control, the experimental group showed higher microbial diversity and more flavor compounds. Starch and acid protease were key physicochemical factors affecting flavor formation, with acid protease positively correlating with major flavors like ethyl palmitate, acetic acid, and octanoic acid. Dominant microbes (*Lactobacillus, Thermoascus, Aspergillus*) in the experimental group were strongly correlated with esters, acids, and alcohols. TTMP content and sensory scores were significantly higher in the experimental group, demonstrating the potential of microbial enhancement to improve baijiu quality.

## Highlights

• A high-TTMP-producing *B. halotolerans* YB13 was obtained by screening and mutagenesis.
• Fortified *Daqu* of *B. halotolerans* YB13 enhances the quality of complex-flavor *Baijiu*.
• Dynamic difference of *Jiupei* microbiota and flavor by the fortified *Daqu* was revealed.
• Flavor correlations decode fortified Daqu's *Baijiu* quality enhancement mechanism.

## 1 Introduction

Chinese baijiu, primarily produced through solid-state fermentation and distillation of cereal grains including sorghum and wheat，has gained global recognition for its distinctive production techniques and complex flavor profiles (Xiao & Bei, 2016). Based on aroma characteristics, Chinese baijiu is conventionally classified into twelve major types, including sauce-aroma, strong-aroma, and light-aroma varieties, among others (Zhang et al., 2018). As an innovative style beyond these traditional categories, Mixed-flavor Baijiu represents a hybrid flavor type that harmoniously combines multiple aromatic attributes. It uniquely integrates the full-bodied complexity of sauce-aroma, the intense fragrance of strong-aroma, and the clean crispness of light-aroma baijiu (Cheng et al., 2023). This distinctive style is typically manufactured through a sophisticated mixed-grain fermentation process, which contributes to its exceptionally rich and multi-layered sensory profile in both aroma and taste (Cheng et al., 2022).

Daqu serves as a crucial saccharification and fermentation agent in Chinese baijiu production due to its rich microbial diversity and enzymatic composition (Allwood et al., 2021). The indigenous microorganisms in bran koji actively participate in synthesizing key flavor compounds, including esters, organic acids, and pyrazines, which are essential for developing baijiu's characteristic aroma profile (Ren et al., 2019). Recent advances in microbial biotechnology have enabled targeted flavor modulation through the strategic inoculation of functional microbial strains into fortified Daqu. Notably, the supplementation of high-ester-yielding yeast strains has demonstrated significant potential for enhancing ester production in Mixed-flavor Baijiu, resulting in improving aromatic complexity and intensity (Yuan et al., 2023).

Tetramethylpyrazine (TTMP), a key flavor compound characteristic of sauce-aroma baijiu, is biosynthesized primarily through microbial metabolic pathways. Recent studies have demonstrated that supplementing bran koji with *B. subtilis* can enhance TTMP content by over 30 % in sauce-aroma baijiu production, while simultaneously improving both aromatic complexity and sensory mouthfeel of law liquor (Shi et al., 2022). Notably, while this microbial augmentation strategy has proven effective for sauce-aroma variants, its application potential remains unexplored in Mixed-flavor Baijiu fermentation process.

The distinctive flavor profile of Chinese baijiu originates from diverse flavor compounds generated through microbial metabolic activities during fermentation, with their formation being intrinsically linked to the microbial communities inhabiting the fermenting grains. Recent advances in flavoromics and microbiome analysis have enabled significant breakthroughs in elucidating microbial-flavor compound correlations across different baijiu aroma types. Comprehensive studies have revealed substantial variations in these microbial-flavor relationships among distinct aroma categories (Liu & Quan, 2024). Notably, a recent metabolomic study identified tetramethylpyrazine (TTMP) as a key biomarker for distinguishing medium-temperature Daqu, underscoring its critical role in shaping Daqu characteristics and, by extension, baijiu flavor (Chen et al., 2025). While current research has established fundamental knowledge regarding fermentation microbiota and their applications, investigations into the temporal dynamics of microbial communities and their interactions with flavor compound evolution remain notably scarce for Mixed-flavor Baijiu—an emerging aroma type—particularly under controlled fermentation conditions. This knowledge gap highlights the need for systematic studies to decipher the complex microbiota-flavor relationships in this novel baijiu variant.

While current research has established fundamental knowledge regarding fermentation microbiota and their applications, investigations into the temporal dynamics of microbial communities and their interactions with flavor compound evolution remain notably scarce for Mixed-flavor Baijiu. This knowledge gap is particularly critical given the unique challenges of this style, such as the stability of mixed-grain fermentation and the complex microbial interactions required for its characteristic flavor (Cheng et al., 2022; Guan et al., 2023). To address this, our study screened for functional strains capable of thriving in this complex system. The obtained *B. halotolerans* YB13, with its high TTMP yield (2.859 mg/g), environmental stress tolerance (Lin et al., 2025), and potential for synergistic metabolism with indigenous microbiota (Tu et al., 2022; Wu, Chen, et al., 2023), presents an ideal candidate for application in fortified Daqu.

Base on microbial strain screening and breeding for high TTMP production, this study systematically analyzed the effects of enriched koji supplementation on dynamic changes of critical fermentation parameters in Mixed-flavor Baijiu production, including physicochemical properties, enzymatic activities, flavor compound profiles, and microbial community. Through comprehensive correlation analysis, the study elucidate the complex interrelationships among these key parameters. By those analysis, this work provides insights into how enriched koji improve flavor in this emerging baijiu variety.

## 2 Materials and methods

### 2.1 Sample collection

High-temperature Daqu samples were collected from a baijiu company in Anhui Province. Fermented grain samples were obtained from five randomly chosen locations at the upper, middle, and bottom layers of each fermentation chamber using a sterile sampling spatula (approximately 100 g per point). The collected subsamples from each layer were thoroughly mixed into a single composite sample, promptly sealed in sterile bags, and maintained in ice-filled containers during transport. Subsequently, all composite samples were stored at −80 °C within two hours post-collection for later determination of physicochemical parameters and microbial community structure. Five random sampling points were selected per fermentation room, and the samples were stored at −80 °C for subsequent experiments.

### 2.2 Screening of TTMP-producing strain

The strain screening method refers to the method of Zhuansun Wanwan (Zhuansun, 2023), the determination of TTMP content after liquid fermentation follows the method of Ding xuemei. (Ding, 2015), and the determination of TTMP content after solid-state fermentation adopts our previous method (Zhuansun, 2023).

### 2.3 Plasma mutagenesis

Strain YB13 was mutagenized following the method described by Zou et al. (2019) with modifications. Briefly, the strain was first activated in LB liquid medium, and the cells were harvested by centrifugation, followed by resuspension in sterile water. Mutagenesis was then performed using an ARTP mutagenesis instrument. The key operating parameters were set as follows: plasma output power of 120 W, helium gas flow rate of 10 SLM (standard liters per minute), and a distance of 2 mm between the plasma torch and the sample. An aliquot of 10 μL of the bacterial suspension was spread evenly onto a sterile stainless steel plate (a dedicated accessory for the ARTP instrument). The plates were subjected to mutagenesis treatment at different time gradients: 0, 20, 40, 60, 80, 100, and 120 s. After each treatment interval, the plate containing the bacterial suspension was entirely transferred into a 1.5 mL sterile EP tube containing 1 mL of sterile physiological saline, followed by vortexing for 2 min for elution. The eluted suspension was then serially diluted, spread onto LB agar plates, and incubated at 37 °C for 48 h to obtain single colonies (Fig. S-1). This protocol allowed for the assessment of the effects of different treatment durations on survival rate and mutation efficiency, facilitating the subsequent screening of positive mutants.

### 2.4 Molecular identification

PCR amplification was performed using the universal primers 27F (5′-AGAGTTTGATCCTGGCTCAG-3′) and 1492R (5′-GGTTACCTTGTTACGACTT-3′). The PCR conditions were as follows:pre-denaturation at 95 °C for 5 min and 25 cycles; 95 °C denaturation for 30 s, 56 °C annealing for 30 s, 72 °C extension for 90 s, exten-sion at 72 °C for 10 min, and storage at 4 °C The amplified sequences were commercially sequenced (Kechuang Biotech) and analyzed using BLAST (NCBI) for homology comparison. Phylogenetic analysis was conducted using MEGA X software with the Neighbor-Joining model.

### 2.5 Fortified Fuqu preparation

The strain *B. halotolerans* YB13 was used for the preparation of fortified Daqu. The inoculum was prepared through a multi-stage seed culture process. Specifically, 10 L of bacterial seed culture was inoculated into a 150 L seed fermenter and cultivated at 45 °C and pH 8.0. This was followed by scaling up to a 1200 L seed fermenter under the same temperature and pH conditions (45 °C, pH 8.0). Subsequently, the 1200 L seed culture was inoculated into a 12-ton disc fermentation system containing wheat bran with a moisture content of 52 %. The disc fermentation was carried out at 50 °C and pH 7.0, while maintaining a consistent moisture content of 52 % throughout the process. This procedure yielded the final fortified Daqu product.

The selection of these process parameters was primarily based on the following considerations. (1) Strain YB13 was isolated from high-temperature Daqu, and its optimal growth temperature range aligns with the process characteristics of high-temperature Daqu. A seed culture temperature of 45 °C is considered favorable for rapid cell proliferation, while a solid-state fermentation temperature of 50 °C was chosen to simulate the high-temperature Daqu fermentation environment, thereby creating an advantageous niche for YB13 that supports efficient metabolism, including TTMP synthesis, while inhibiting the growth of most contaminants. (2) A pH of 8.0 for the seed culture was selected based on the general understanding that bacteria of the genus *Bacillus* typically exhibit more vigorous growth in neutral to slightly alkaline conditions. An initial pH of 7.0 for solid-state fermentation was maintained as it is closer to the natural pH of the bran raw material, potentially favoring synergistic interactions within the microbial community. (3) A moisture content of 52 % was used as it falls within the common range for Daqu preparation, ensuring sufficient water availability for microbial growth without causing excessive wetness.

### 2.6 Fermentation sampling

The Fortified-Daqu mixtures were piled for 72 h, with samples collected at 0, 24, 48, and 72-h intervals. After the 72-h culture period, the mixtures were transferred to fermentation pits for brewing. Both experimental (inoculated with strain YB13) and control groups were sampled at days 1, 4, 7, 12, 17, 27, 37, and 57. Sampling was conducted from upper, middle, and lower pit layers, with five replicates collected per layer (Fig. S-2). All samples were immediately stored at −80 °C for subsequent analysis.

### 2.7 Physicochemical analysis

Moisture content (by loss-on-drying at 105 °C), acidity (by acid-base titration, expressed as lactic acid), reducing sugar content (by the DNS method using a glucose standard), and starch content (by acid hydrolysis followed by reducing sugar quantification) were analyzed according to the DB34/T 2264–2014 standard method and the cited DNS protocol (Miller, 1959; Weng, 2019).

### 2.8 Enzyme assays

The activities of amylase and glucoamylase were determined using the DNS method (Miller, 1959). Acid protease activity was measured via the Folin-phenol assay, while cellulase activity was assessed using the carboxymethyl cellulose (CMC) hydrolysis method (Wang, 2015).

### 2.9 TTMP quantification

Those samples obtained from both liquid fermentation and solid-state fermentation was pretreated using the method described by Zhuansun et al. (Zhuansun, 2023). TTMP content was quantified according to Xue et al.'s method employing HS-SPME-GC–MS with selected ion monitoring (SIM) mode calibration (Xue et al., 2024). Method validation confirmed excellent linearity within the tested concentration range, with a coefficient of determination (R^2^) of 0.9991. The limit of detection (LOD) and limit of quantification (LOQ) for TTMP were determined to be 3.0 μg/kg and 10.0 μg/kg, respectively, demonstrating high sensitivity and reliability of the analytical method.

### 2.10 Volatile profiling

Volatile compounds in fermented grains were analyzed following an optimized HS-SPME-GC–MS protocol adapted from Dai et al. (Dai et al., 2024). Briefly, 10 g of fermented grain sample was homogenized with 1 % anhydrous CaCl₂ and 20 mL of boiled ultrapure water, then refrigerated at 4 °C overnight. The mixture underwent ultrasonication in an ice-water bath for 30 min followed by centrifugation (10,000 *g, 20 min, 4 °C) to obtain the supernatant. For analysis, 5 mL of supernatant was transferred to a 20 mL headspace vial containing 3 g NaCl and internal standard (n-amyl acetate, 86.68 μg/L final concentration).

The SPME procedure utilized a tri-phase fiber (DVB/CAR/PDMS, 50/30 μm) with the following parameters: 50 °C initial temperature, 5 min pre-equilibration, 45 min extraction, and 5 min desorption. Chromatographic separation was achieved using a DB-FFAP capillary column (60 m × 0.25 mm × 0.25 μm) with temperature programming: 50 °C (hold 2 min), then 6 °C/min ramp to 230 °C (hold 15 min). The GC system operated with helium carrier gas (2 mL/min) in splitless mode, with injector and detector temperatures maintained at 250 °C.

Mass spectrometric detection employed electron impact ionization (70 eV) with an ion source temperature of 230 °C and mass scanning range of 35–350 amu. Quantification was performed using external calibration curves prepared for each target compound.

It is important to acknowledge the inherent limitations of the HS-SPME-GC–MS method used in this study for the quantitative analysis of volatile flavor compounds. The selectivity of the SPME fiber coating toward compounds of specific polarities and molecular sizes can result in preferential enrichment of certain analytes and under-representation of others. Furthermore, in complex matrices such as fermented grains, competitive adsorption among volatile compounds may affect extraction efficiencies, and matrix effects can also influence quantitative accuracy.

Although measures were implemented to minimize these biases—including optimization of extraction parameters, the use of an internal standard (n-amyl acetate), and calibration with external standard curves—the absolute quantitation of volatile components may still be affected by the aforementioned factors. Therefore, the concentration data presented in this study should be interpreted as relative quantitative values obtained under specific methodological conditions. They are primarily applicable for assessing comparative trends in flavor compound abundance across samples, rather than as absolute concentrations.

### 2.11 Microbial community analysis of fermented grains

Total genomic DNA was extracted from Jiupei samples using the DNA Isolation Kit (Zhang et al., 2021). DNA concentration, purity, and integrity were evaluated through PicoGreen fluorometric quantification and agarose gel electrophoresis. Qualified DNA samples were normalized to 20 ng/μL for downstream applications (Wang, 2021).

The V3-V4 hypervariable regions of bacterial 16S rRNA genes were PCR-amplified using universal primers 338F (5’-ACTCCTACGGGAGGCAGCAG-3′) and 806R (5’-GGACTACHVGGGTWTCTAAT-3′). Fungal ITS1 regions were amplified with primers ITS5-1737F (5’-GGAAGTAAAAGTCGTAACAAGG-3′) and ITS1R (5’-GCTGCGTTCTTCATCGATGC-3′). All samples (including both bacterial and fungal communities) were subjected to high-throughput sequencing using the Illumina Novaseq 6000 platform, PE250 mode. This platform provides read length, throughput, and accuracy suitable for the microbial community structure analysis required by this study. Sequencing quality parameters were: Q30 ≥ 80 %, effective read length approximately 450 bp (after removing primers and barcodes), ensuring data reliability. All samples underwent the same library preparation and sequencing procedures to minimize technical bias and ensure comparability between bacterial and fungal data.

Following sequencing, raw reads underwent quality filtering, sequence assembly, and operational taxonomic unit (OTU) clustering. Microbial α-diversity was assessed using Chao1, Ace, Shannon, and Simpson indices. For taxonomic classification, representative OTU sequences were aligned against the SILVA (Version 138) database for bacteria and the UNITE (Version 8.3) database for fungi (Mou, 2015).

### 2.12 Sensory evaluation

Sensory evaluation was performed by four trained senior panelists from the Technology Center of Anhui Jinzhongzi Distillery Co., Ltd. All panelists held nationally recognized certifications in baijiu sensory assessment and possessed over five years of professional tasting experience. Evaluations were conducted in a controlled sensory analysis laboratory.

Base liquor samples from both the experimental group (A-series: A-Top, A-Middle, A-Bottom, A-Mixed) and the control group (C-series: C-Top, C-Middle, C-Bottom, C-Mixed) were assessed, covering stratified (upper, middle, lower) and blended layers. A 100-point scoring system was employed to evaluate key sensory attributes, including cellar aroma intensity, aroma harmony, initial sweetness and smoothness, taste fullness, cleanness of aftertaste, and typical style. Each sample was assessed in three independent tasting replicates by every panelist. The final sensory scores were calculated as the mean of all evaluations across all panelists and replicates to minimize individual variability and random errors, thereby ensuring the reliability and robustness of the data.

### 2.13 Statistics

All measurements were conducted in triplicate and expressed as mean ± SD. Data were visualized using Origin 2021, while statistical analysis (*p* < 0.05) was performed with SPSS 21.0. Flavor compounds were analyzed by GC–MS Solution v2.10, and redundancy analysis (RDA) was conducted using Canoco 4.5.

## 3 Results

### 3.1 Screening the strain displaying high producing TTMP in soild-state fermentation

Seventy-two bacterial strains were isolated from high-temperature Daqu samples using dilution plating. Subsequently, 50 thermotolerant bacterial strains capable of optimal growth at 50 °C were selected through high-temperature screening. Among these, 36 strains showed TTMP production potential based on V—P tests and liquid fermentation (Table S1-S2). Solid-state fermentation further identified 18 TTMP-producing strains, with one strain exhibiting the highest TTMP content (1.366 mg/g, Tab. S-3).

The top-producing strain underwent plasma mutagenesis, generating 36 mutants. Subsequent liquid and solid-state fermentation screening revealed mutant YB13 as the highest TTMP producer with 2.859 mg/g (Table S-4), representing a 2.1-fold increase over the wild-type strain.

### 3.2 Phylogenetic analysis

**Fig. 1:**
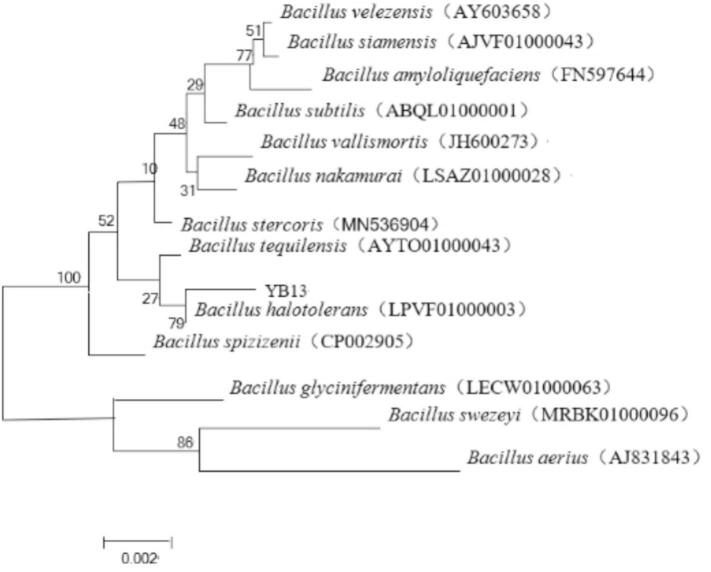
Phylogenetic tree of strain YB13 based on 16S rDNA sequence analysis.

Through 16S rDNA sequence analysis (Fig. 1), strain YB13 showed 99.52 % similarity with *B. halotolerans* and was accordingly identified as *B. halotolerans* YB13. The strain has been deposited in GenBank under accession number PQ303489. *B. halotolerans* was stored at the General Microbial Culture Preservation Management Center of China (CGMCC no. 28161).

### 3.3 Changes in physicochemical parameters of fermented grains mash

**Fig. 2:**
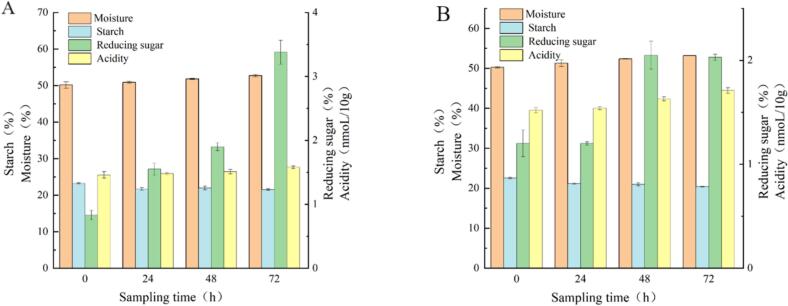
Changes in physicochemical indicators during the stacking process of grain ferment: A) Experimental group; B) Control group.

During grain mash piling, comparative analysis revealed distinct metabolic profiles between the experimental and control groups (Fig. 2-A). The experimental group demonstrated a 5.12 % moisture content increase, 7.63 % starch reduction, remarkable 307.23 % elevation in reducing sugars, and 8.23 % acidity rise (Fig. 2-A). In contrast, the control group showed higher moisture (5.81 %) and acidity (12.5 %) increases, greater starch degradation (9.62 %), but substantially lower reducing sugar accumulation (69.16 %) (Fig. 2-B). The results indicate that the experimental group exhibited higher reducing sugar production efficiency and relatively lower acidity increase during the grain-fermentation mash piling process, demonstrating stronger saccharification potential. This may contribute to improved alcohol yield and enhanced accumulation of flavor compounds. Therefore, the experimental group is more suitable for the production of high-quality liquor with rich flavor and smooth taste. These results indicate that the inoculation of YB13 via fortified Daqu potentially enhances the saccharification capacity and regulates acid metabolism of the system, thereby providing a more favorable fermentation microenvironment for subsequent flavor formation.

### 3.4 Changes in physicochemical parameters of fermented mash

**Fig. 3:**
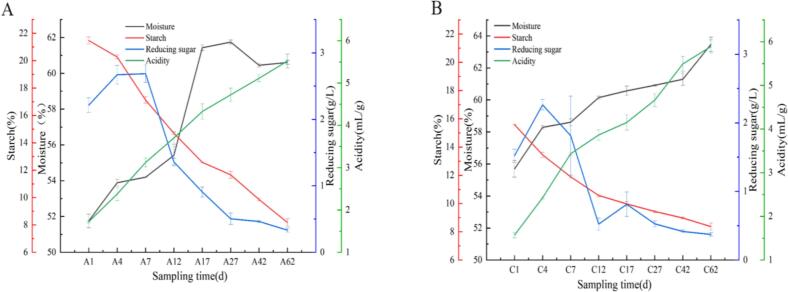
Changes in physicochemical indicators of fermented grains during the brewing process: A) Experimental group; B) Control group.

During the fermentation process of the experimental group with adding fortified Daqu, water content gradually increased from 51.75 ± 0.38 to 60.61 ± 0.11, representing a 17.12 % increase. A continuous reduction of 61.90 % was observed in starch content, decreasing from 21.47 ± 0.26 to 8.18 ± 0.28 (Fig. 3-A). The reducing sugar content decreased from 2.21 ± 0.11 to 0.33 ± 0.03, representing an 85.07 % reduction. Meanwhile, the acidity increased significantly from 1.72 ± 0.02 to 5.52 ± 0.17, representing a 220.93 % rise (Fig. 3-A).

In the control group, physicochemical parameters exhibited similar trends to the experimental group. Moisture content increased by 13.97 % (from 55.69 ± 0.51 to 63.47 ± 0.45), while starch content decreased by 46.28 % (15.58 ± 0.02 to 8.37 ± 0.25). Reducing sugars showed a 75.66 % reduction (1.52 ± 0.09 to 0.37 ± 0.03), and acidity increased dramatically by 275.16 % (1.57 ± 0.06 to 5.89 ± 0.15) (Fig. 3-B).

Elevated moisture levels facilitate starch hydrolysis, producing reducing sugars that fuel microbial growth and metabolism. Concurrently, increasing acidity modulates microbial activity by inhibiting undesirable microorganisms, thereby ensuring proper fermentation (Sun et al., 2021). Notably, the enriched koji group demonstrated enhanced starch degradation and more rapid reducing sugar consumption, indicating its potential for producing baijiu with higher alcohol content and more complex flavor profiles.

### 3.5 Changes in enzyme activities during fermentation

**Fig. 4:**
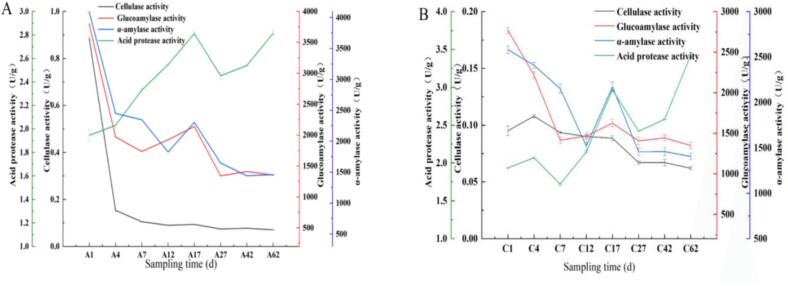
Changes in enzyme activity of fermented grains during the brewing process: A) Experimental group; B) Control group.

In the experimental group, enzymatic activities in the fermented grains exhibited distinct patterns during fermentation (Fig. 4-A). Cellulase activity demonstrated a progressive decline from 0.89 ± 0.01 U/g to 0.07 ± 0.00 U/g, achieving a remarkable 92.04 % reduction. Both glucoamylase and α-amylase activities followed decreasing trends, with respective reductions of 64.35 % (3796.60 ± 5.02 to 1353.62 ± 0.29 U/g) and 64.29 % (4089.00 ± 0.89 to 1460.37 ± 0.31 U/g). Notably, acid protease activity displayed a characteristic fluctuation pattern, featuring an initial increase followed by decline and subsequent recovery, peaking on day 17. The control group mirrored similar but less pronounced trends in enzymatic changes (Fig. 4-B). Cellulase activity was reduced by 40.00 % (0.10 ± 0.00 to 0.06 ± 0.00 U/g), while glucoamylase and α-amylase showed decreases of 51.34 % (2770.32 ± 0.59 to 1347.96 ± 0.63 U/g) and 45.57 % (2581.68 ± 1.78 to 1405.33 ± 0.84 U/g), respectively. Contrasting with the experimental group, acid protease activity in controls maintained a steady increasing trajectory, attaining maximum activity on day 62. The experimental group exhibited strong starch degradation capacity and a higher rate of reducing sugar production during the early fermentation stage, providing sufficient carbon sources for the subsequent synthesis of alcohol and flavor compounds. Meanwhile, the dynamic changes in acid protease activity, together with the synergistic effect of the enhanced qu starter, further enhanced the metabolic advantages of the experimental group in flavor compound biosynthesis (Wu, Duan, et al., 2023). The differences in enzyme activity dynamics, particularly the higher amylolytic activity and the distinct fluctuation pattern of acid protease in the experimental group, suggest that the addition of YB13 reshapes the metabolic functionality of the fermentation system, which may directly influence precursor supply and the synthesis rate of flavor compounds.

### 3.6 Changes in TTMP content in fermented grains and mash

**Fig. 5:**
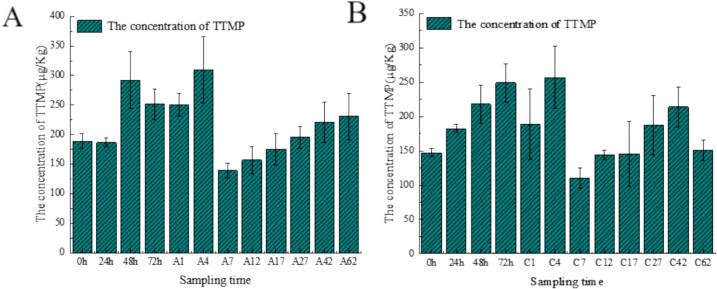
Changes in TTMP content in Fortified Daqu (0-72 h) and distiller's grains (A1–72 and C1–72). A: Experimental Group B: Control Group.

Fig. 5-A demonstrates the dynamic changes in TTMP content during the fermentation process in the experimental group. During the grain mash piling stage (0–48 h), TTMP levels showed a progressive increase, peaking at 48 h before experiencing a modest decline by 72 h. In the subsequent fermentation phase, TTMP content exhibited a biphasic pattern: an initial increase culminating in a maximum concentration on day 4, followed by a sharp decrease on day 7 - potentially attributable to microbial community dynamics or variations in fermentation parameters. Notably, from day 7 through day 62, a gradual recovery in TTMP content was observed.

Fig. 5-B illustrates the control group's TTMP profile, which displayed generally comparable trends to the experimental group, albeit with quantitative differences. As a key flavor-active compound, TTMP significantly contributes to the sensory characteristics of baijiu. Previous studies have established that *B.* spp. are primarily responsible for TTMP biosynthesis (Wu et al., 2023). This study demonstrated that the experimental group with added enhanced qu starter exhibited higher TTMP content than the control group during both grain mash piling and fermentation processes, indicating a stronger capacity for flavor compound synthesis. In addition, the increased TTMP levels may potentially confer greater health benefits to the final liquor product.

### 3.7 Changes in flavor compound composition of fermented mash

**Fig. 6:**
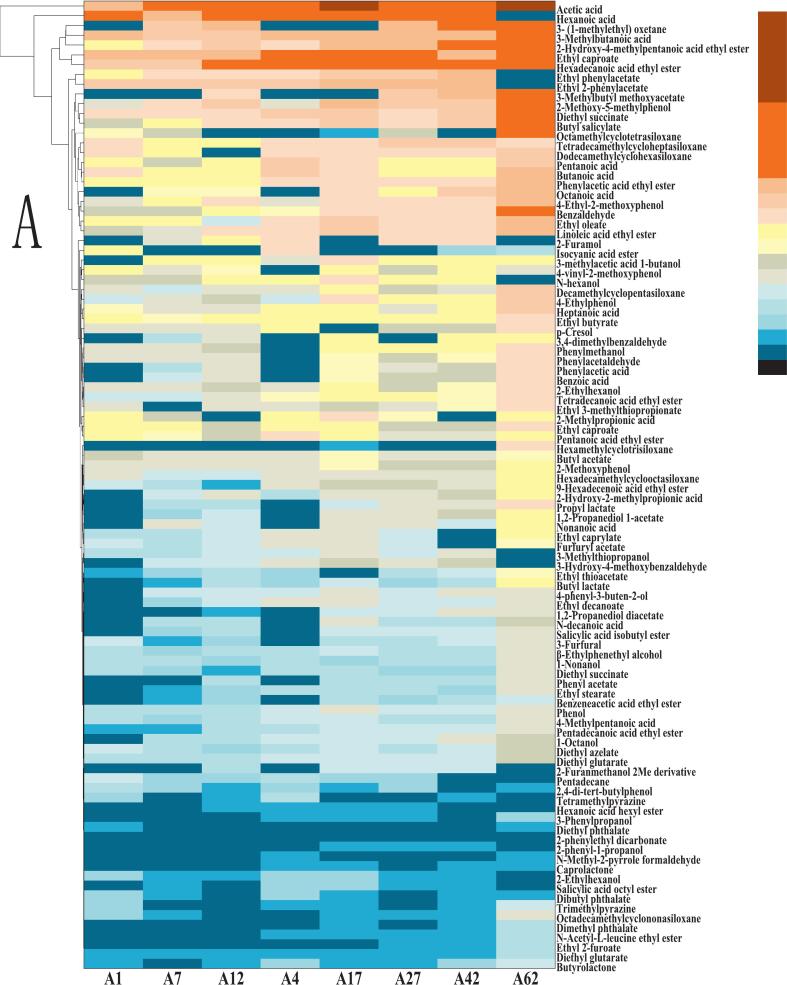

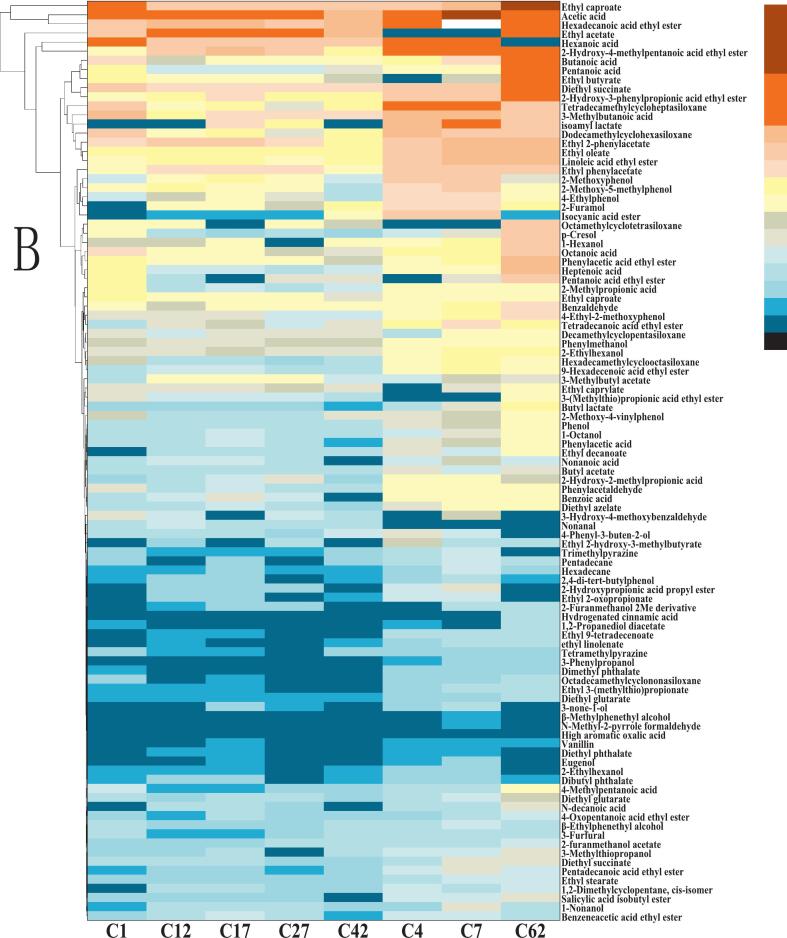
Flavor compounds in fermented grains during solid-state fermentation: A) Experimental group; B) Control group.

As indicated in Fig. 6-A, Fig. 6-B，Tab. S-5，and S-6, esters constitute the predominant class of flavor compounds in baijiu, playing a pivotal role in defining its sensory characteristics. These compounds are primarily synthesized via esterification reactions between alcohols and organic acids during fermentation and are known to impart desirable fruity and floral aromas, which are fundamental to the spirit's overall flavor profile (Lytra et al., 2012). In the experimental group (enriched-koji), the major esters detected include ethyl palmitate, ethyl myristate, ethyl linoleate, ethyl 3-phenylpropanoate, ethyl salicylate, ethyl hexanoate, diethyl succinate, ethyl oleate, ethyl 2-hydroxy-4-methylpentanoate, and ethyl phenylacetate. The control group exhibited a similar ester profile, with key compounds such as ethyl palmitate, ethyl linoleate, ethyl pentanoate, ethyl phenylpropanoate, isopentyl lactate, ethyl acetate, diethyl succinate, ethyl oleate, ethyl butyrate, and ethyl phenylacetate. Notably, the experimental group demonstrated a higher concentration of ethyl myristate (11.07 mg/kg) compared to the control (9.34 mg/kg). Ethyl myristate is known to enhance the sweetness and aromatic smoothness of baijiu, contributing to its flavor complexity. Studies suggest that this ester is particularly abundant in baijiu compared to other distilled spirits, playing a key role in its perceived sweetness (Lytra et al., 2012). the presence of ethyl 2-hydroxy-4-methylpentanoate in the experimental group further enriched the fruity aromatic characteristics, playing a positive role in improving the overall aroma complexity and sensory quality of the liquor. (Wang et al., 2024).

Acidic compounds are crucial in shaping baijiu's flavor profile, influencing taste balance, enhancing sweetness, and contributing to aromatic complexity. The experimental group was characterized by dominant acids such as acetic acid, 3-methylbutyric acid, hexanoic acid, butyric acid, and octanoic acid, while the control group additionally contained a relatively high level of pentanoic acid.

Aldehydes significantly impact baijiu's flavor through their distinct sensory properties, volatility, and interactions with other compounds. Both experimental and control groups contained key aldehydes, including benzaldehyde, 3-hydroxy-4-methoxybenzaldehyde, and phenylacetaldehyde. These compounds not only define the spirit's aromatic profile but also enhance its mouthfeel, adding depth and layering to the drinking experience.

The experimental group featured prominent alcohols such as 1-nonanol, 2-phenyl-1-propanol, 2-ethylhexanol, and 4-phenyl-3-buten-2-ol, whereas the control group was dominated by 1-hexanol, benzyl alcohol, and 2-furanmethanol. These alcohols contribute to the overall complexity and richness of baijiu's flavor.

Studies have shown that although phenolic compounds are present in relatively low concentrations in Baijiu, they play a significant role in shaping its flavor profile. Even trace components can exert key effects on olfactory and gustatory perception, thereby enhancing the complexity and depth of the liquor's sensory characteristics (Ni et al., 2024). In the experimental group, the predominant phenolic compounds included 2,4-di-tert-butylphenol, 4-ethylphenol, and phenol, which are typically associated with more intense and persistent aromatic properties, indicating their potential to improve the overall sensory quality of Baijiu. In contrast, the control group was mainly characterized by phenolic compounds such as 2-methoxy-4-vinylphenol, 4-ethyl-2-methoxyphenol, 2-methoxy-5-methylphenol, p-cresol, and 2-methoxyphenol, which generally exhibit milder aroma profiles. Compared with the control group, the relative enrichment of specific active phenolic compounds in the experimental group demonstrates a stronger capacity for flavor modulation, further supporting its advantages in enhancing flavor complexity and sensory quality in Baijiu production.

Alkanes contribute to baijiu's aroma, taste, and overall flavor balance. The experimental group contained octamethylcyclotetrasiloxane, dodecamethylcyclohexasiloxane, tetradecamethylcycloheptasiloxane, and 3-(1-methylethyl) oxetane, while the control group shared the first three alkanes but lacked the latter.

Analysis of the dynamic changes in flavor compounds during fermentation (Tab. S-5, S-6) revealed a clear advantage for the experimental group in the synthesis and accumulation of flavor substances. Relative to the initial fermentation stage (Day 1), several key esters in the experimental group, such as ethyl palmitate and ethyl myristate, exhibited stronger accumulation trends during the mid to late stages. Notably, the concentration of ethyl myristate at the mature fermentation stage was 1.2 times greater in the experimental group than in the control. Furthermore, a unique fruity ester, ethyl 2-hydroxy-4-methylpentanoate, was generated and steadily accumulated from the mid-fermentation stage onward in the experimental group but was not detected in the control. These observed trends demonstrate that the introduction of YB13-fortified Daqu quantitatively enhanced the yield of specific flavor compounds and potentially activated new flavor synthesis pathways. The positive changes observed during fermentation, relative to both the initial state and the control group, clearly demonstrate the optimizing effect of YB13-fortified Daqu on the flavor metabolic network, i.e., promoting the generation and sustained accumulation of specific flavor compounds (especially esters). This provides a direct material basis for the ultimately enhanced sensory quality of the experimental group's base liquor.

### 3.8 Principal component analysis of flavor compounds in fermented mash

**Fig. 7:**
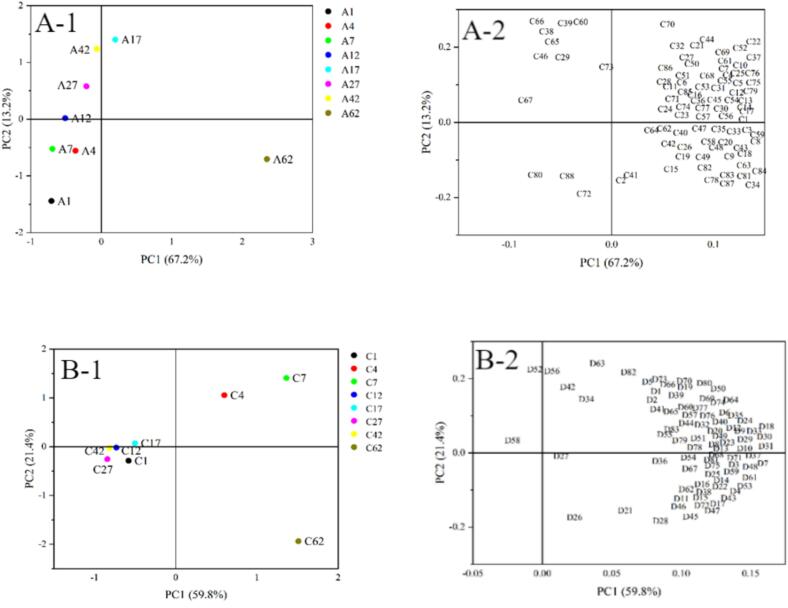
Score and loading plots of fermented grain samples during the fermentation process: A) Experimental group; B) Control group.

Multivariate statistical analysis was conducted on flavor compounds detected at a frequency ≥ 80 % in fermented grains (Tab S-7，S-8) to characterize the temporal evolution of flavor profiles throughout fermentation. Principal component analysis (PCA) was employed, with results visualized through score and loading plots (Fig. 7).

The experimental group (A-1) demonstrated well-defined fermentation dynamics through its volatile compound distribution pattern, with temporal evolution clearly demarcating three distinct phases: early (days 1–7, positioned below X-axis), middle (days 12–42 clustered near Y-axis above X-axis), and late stage (day 62). Principal component analysis (Figure A-2) identified characteristic markers for each phase: quadrant III contained early-stage volatiles (C80 pentadecane, C88 tetramethylpyrazine, C72 2,4-di-tert-butylphenol), while other quadrants harbored mid- and late-stage signature compounds. In contrast, the control group (B-1) exhibited less coherent clustering, requiring division into four phases (initial, early-middle, late, and terminal) with discontinuous flavor transitions, notably featuring day 17's unique markers (D58 3-methyl-1-butanol acetate, D52 *N*-methyl-2-pyrrolecarboxaldehyde) in quadrant II. Comparative evaluation revealed the experimental group's superior fermentation characteristics: (1) clearer phase demarcation, (2) more concentrated flavor compound generation, and (3) regular temporal progression. This systematic flavor development contrasts sharply with the control's dispersed dynamics and discontinuous compound formation, which compromised flavor layering and stability. The results provide empirical evidence for the technological advantages of the fortified starter system in achieving enhanced flavor consistency and complexity in premium baijiu production.

### 3.9 Microbial diversity dynamics during baijiu fermentation

#### 3.9.1 Bacterial diversity changes

The bacterial diversity dynamics in fermented grains during the fermentation process were comprehensively analyzed using established ecological indices. Microbial richness was assessed via the Chao1 and Observed Species indices, while diversity was evaluated using the Shannon and Simpson indices. Sequencing depth was verified by the Coverage index. These metrics collectively characterized the microbial community composition of the fermented grain samples, with detailed results presented in Table S-9.

As presented in Tab. S-9, the experimental group displayed pronounced bacterial richness and diversity during the initial fermentation stage (Day 1). A marked reduction was observed by Day 4, likely due to early-stage fermentation dynamics or environmental constraints. However, a significant rebound occurred by Day 7. From Day 7 onward, both richness and diversity exhibited a gradual yet consistent decline until Day 62. The control group followed a broadly similar trend, though the experimental group maintained superior microbial richness and diversity in the early phase. This enhanced microbial activity in the initial stage may have facilitated the development of flavor precursors, thereby supporting the formation of complex flavor profiles in later fermentation stages.

#### 3.9.2 Fungal diversity changes

High-throughput sequencing was employed to characterize the fungal diversity and community composition throughout the fermentation process of the grain substrate. Analysis revealed excellent sequencing coverage across all samples, with Goods coverage values consistently exceeding 0.9999, indicating comprehensive representation of the fungal communities. Complete analytical results are provided in Table S-10.

As summarized in Tab.S-10, fungal richness and diversity in the experimental group peaked on day 7 of fermentation. Subsequently, these parameters exhibited a gradual decline until day 27, followed by a resurgence on day 42. In contrast, the control group demonstrated a consistent upward trajectory, achieving maximal values by day 62. Comparative analysis indicated that the experimental group maintained statistically superior performance across all measured ecological indices, including community richness (Chao1), diversity (Shannon), evenness (Pielou), and sequencing coverage (Goods). This enhanced fungal community structure in the experimental group likely contributed to more robust metabolic activity, thereby facilitating the biosynthesis of diverse flavor precursors. Consequently, the experimental group demonstrated superior organoleptic qualities, as evidenced by enhanced aromatic complexity, improved taste profile stratification, and greater liquor body stability relative to the control.

### 3.10 Microbial community structure during fermentation

#### 3.10.1 Bacterial community analysis

**Fig. 8:**
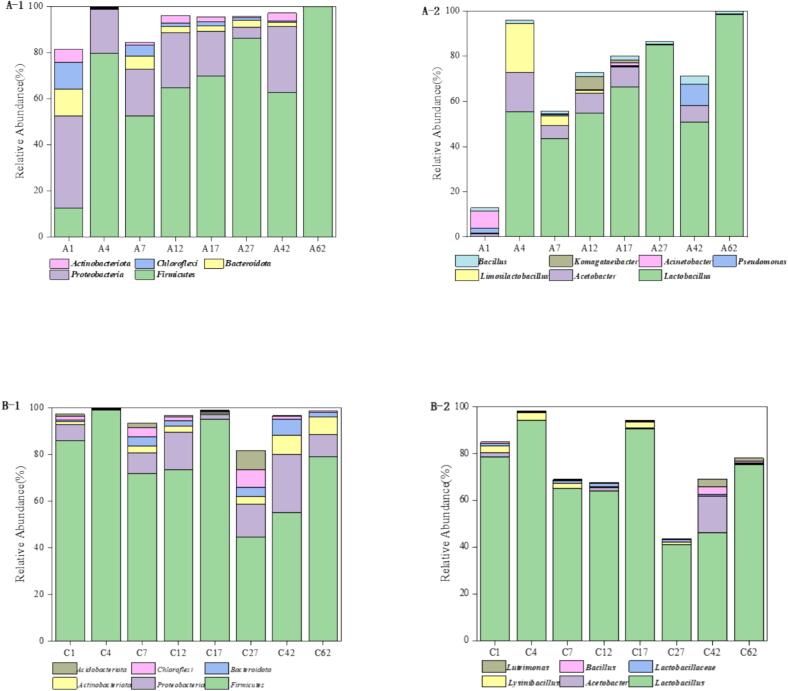
Dynamics of the relative abundance of major bacterial phyla and genera in fermented grains during the fermentation process: A) Experimental group; B) Control group. Note A1 and B1 present phylum level, A2 and B2 prsent genus level.

Microbial community analysis revealed significant differences between experimental and control groups (Fig. 8). In the experimental group (Fig. 8A-1), Firmicutes dominated as the predominant bacterial phylum throughout fermentation, maintaining >50 % relative abundance after day 1 and ultimately reaching 99.9 % by day 62, showing a consistent increasing trend. Proteobacteria served as the secondary phylum (average > 4 % from day 1–42) but exhibited gradual decline despite fluctuations. At genus level (Fig. 8A-2), Lactobacillus emerged as dominant from day 4 onward, achieving 98.61 % relative abundance by day 62 (average > 44 %). The control group (Fig. 8B-1) showed similar phylum-level composition (Firmicutes and Proteobacteria), but Firmicutes displayed an overall decreasing trend with notable declines on days 7 and 27 before partial recovery, despite higher initial abundance than the experimental group. Genus-level analysis (Fig. 8B-2) confirmed Lactobacillus dominance but with lower stability. Comparative analysis demonstrated the experimental group's superior microbial profile, characterized by significantly higher and more stable abundances of both Firmicutes and Lactobacillus, particularly during mid-late fermentation stages. This stable consortium, including consistently abundant *B.* populations, promoted efficient fermentation and enhanced flavor/aroma complexity. Conversely, the control group's fluctuating community structure, particularly in Firmicutes populations, may disrupt critical metabolic pathways (including TTMP production), potentially compromising final product quality and sensory balance. These findings underscore the importance of microbial community stability for optimal baijiu fermentation outcomes.

#### 3.10.2 Analysis of fungal community structure

**Fig. 9:**
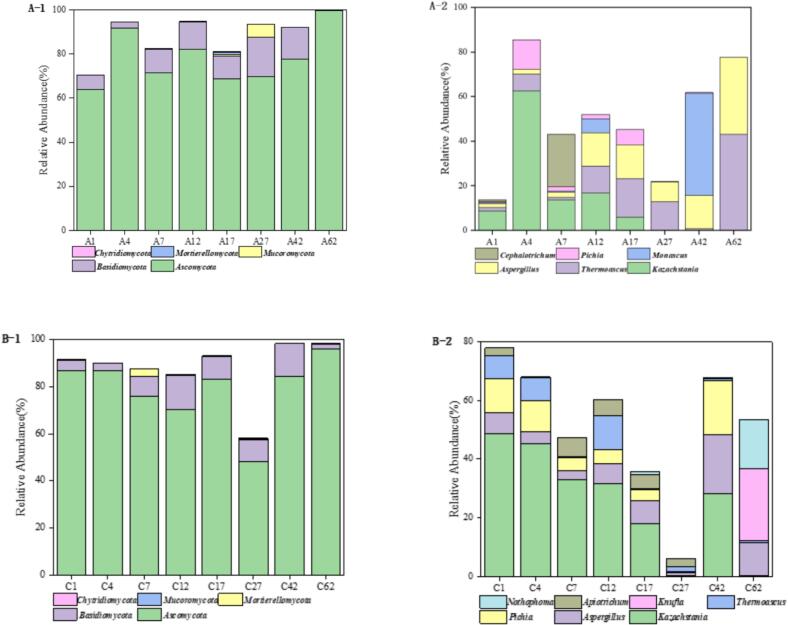
Dynamics of the relative abundance of major fungal phyla and genera in fermented grains during the fermentation process: A) Experimental group; B) Control group. Note A1 and B1 present phylum level, A2 and B2 prsent genus level.

Fungal community analysis revealed distinct compositional differences between experimental and control groups (Fig. 9). In the experimental group (Fig. 9A-1), Ascomycota and Basidiomycota dominated the fungal phyla, with Ascomycota showing increasing predominance throughout fermentation while Basidiomycota maintained stable relative abundance (0.02–18.08 %). At the genus level (Fig. 9A-2), *Kazachstania* dominated initial fermentation stages, with Cephaliotrichum emerging as co-dominant by day 7. From day 12–62, the community diversified to include *Kazachstania*, *Thermoascus*, *Aspergillus*, and *Monascus* as primary genera. The control group (Fig. 9B-1) showed similar phylum-level dominance by *Ascomycota* and *Basidiomycota*, but genus-level analysis (Fig. 9B-2) revealed less diversity, with *Kazachstania* maintaining overwhelming dominance alongside minor contributions from *Aspergillus* and *Pichia*. Comparative analysis demonstrated the experimental group's superior microbial profile, characterized by significantly higher relative abundances of Ascomycota and functional genera (*Kazachstania*, *Thermoascus*, *Aspergillus*, and *Monascus*). This enriched consortium promoted more efficient and stable fermentation, ultimately enhancing flavor harmony and aromatic complexity in the final product. In contrast, the control group's limited microbial diversity and unstable community structure likely contributed to imbalanced flavor compound production, negatively impacting sensory quality. These findings highlight the critical relationship between microbial community structure and baijiu quality parameters. The significant differences in microbial community structure indicate that the successful colonization of YB13 not only exerts its own effects but likely acts as a key driver, reconstructing the microbial interaction network during fermentation, thereby selectively enriching functional genera such as *Lactobacillus*, *Thermoascus*, and *Aspergillus*, and forming a more stable and efficient functional consortium.

### 3.11 Correlation analysis between physicochemical parameters and flavor compounds

**Fig. 10:**
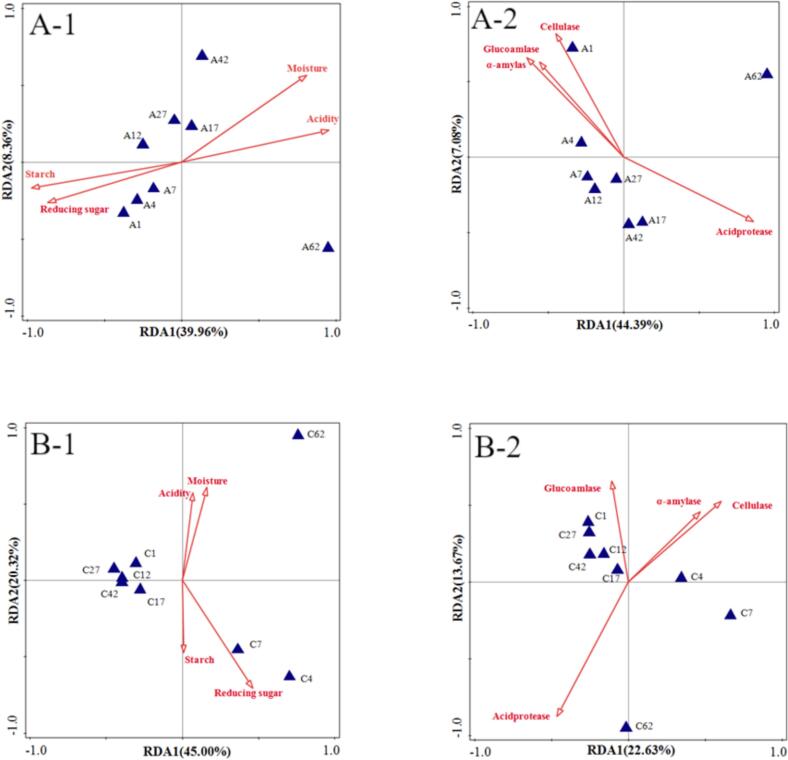
Redundancy Analysis (RDA)plot of physicochemical indicators and flavor compounds in fermented grains during the brewing process: A) Experimental group; B) Control group.

Redundancy analysis (RDA) combined with Pearson correlation coefficientsrevealed significant influences of physicochemical factors on flavor compounds. Fig. 10-A demonstrates strong correlations between physicochemical indices and flavor substances, with the two principal axes explaining 39.96 % and 44.39 % of total variance respectively. Notably, starch and acid protease emerged as predominant factors affecting flavor profiles. Correlation analysis (Table S-11) indicated moisture content showed significant associations (*p* < 0.05) with 16 flavor compounds, while starch, reducing sugars, and acidity correlated significantly with 31, 19, and 31 compounds respectively. Enzymatic influences were also observed, with cellulase, glucosidase, and α-amylase showing significant correlations with 4, 1, and 4 flavor compounds respectively, while acid protease correlated with 24 compounds. Comparative analysis of the control group (Fig. 10-B) revealed physicochemical indices explained 45.00 % and 22.63 % of flavor compound variance, with moisture being the dominant factor. Table S-12 demonstrates control group moisture, starch, and acidity significantly correlated with caproic acid (*p* < 0.05), while reducing sugars showed associations with 9 flavor compounds. Enzymatic correlations were less pronounced in controls, with cellulase, glucoamylase, α-amylase, and acid protease showing significant relationships with 3, 1 (ethyl heptanoate), 2, and 7 flavor compounds respectively. Collectively, these results demonstrate stronger and more numerous associations between physicochemical indices and major flavor compounds in the experimental group, particularly regarding acceptance-related parameters.

### 3.12 Correlation analysis between physicochemical parameters and microbial communities

**Fig. 11:**
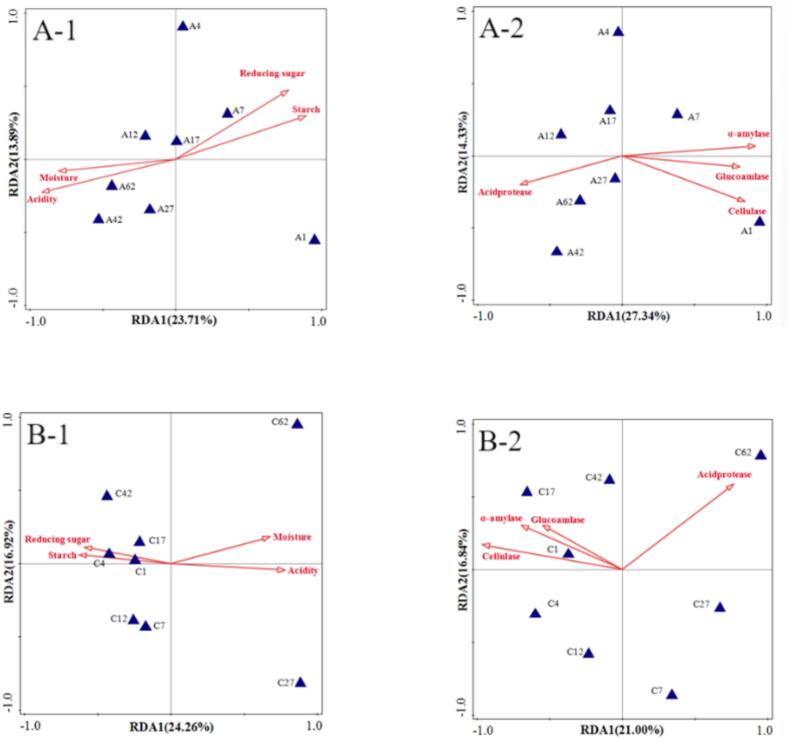
RDA (Redundancy Analysis) plot illustrating the relationships between physicochemical indicators and microbial community in fermented grains during the brewing process: A) Experimental group; B) Control group.

Pearson correlation analysis revealed significant relationships between physicochemical parameters and microbial communities in fermented grains (Tab. S-11, S12). In the experimental group (Fig. 11-A), physicochemical factors accounted for 23.71 % and 27.34 % of microbial variation, with acidity and α-amylase activity emerging as the principal determinants. Analysis demonstrated that moisture content positively correlated with Lactobacillus abundance (*p* < 0.05), whereas starch content exhibited negative correlations with both *Lactobacillus* and *Aspergillus* (*p* < 0.05). Reducing sugars showed an inverse relationship with *Aspergillus* (*p* < 0.05), while acidity positively correlated with *Lactobacillus* and *Aspergillus* but negatively with *Methylobacterium* (*p* < 0.05). Enzymatic activity profiles indicated that cellulase and glucoamylase each correlated with eight bacterial genera, α-amylase with nine, and acid protease with two. In the control group (Fig. 11-B), physicochemical parameters explained 24.26 % and 21.00 % of microbial variation, primarily influenced by cellulase activity. Here, moisture content negatively correlated with *Lactobacillus*, *Kazachstania*, and *Saccharomyces* (*p* < 0.05), contrasting with positive correlations observed for starch content with these same genera. Reducing sugars showed positive associations with *Lactobacillus* and *Kazachstania* (*p* < 0.05), while acidity displayed negative correlations. Both cellulase and α-amylase activities positively correlated with *Lactobacillus* and *Kazachstania* (*p* < 0.05), with glucoamylase additionally associating with *Saccharomyces*. Acid protease activity demonstrated significant correlations with seven bacterial genera.

### 3.13 Correlation analysis between flavor compounds and microbial communities during fermentation

Pearson correlation analysis was conducted to evaluate the relationships between flavor compounds and microbial communities in fermented grains, with detailed correlation coefficients presented in Tables S10 and S11.

Statistical analysis of microbial-flavor compound correlations (Table S10) revealed distinct patterns between experimental and control groups. In the experimental group, ester compounds demonstrated significant positive correlations with seven microbial genera and negative correlations with nine microbial genera (*p* < 0.05). Similarly, acids showed positive correlations with six genera and negative correlations with three, while aldehydes correlated negatively with two microbial genera and positively with three. Alcohols exhibited positive correlations with four genera and negative correlations with seven, and phenolics showed positive correlations with four genera. Notably, the dominant genera *Thermoascus*, *Aspergillus*, and *Lodderomyces* displayed particularly strong associations with flavor compounds, suggesting their pivotal role in flavor development throughout fermentation.

In contrast, the control group exhibited different correlation patterns: esters correlated positively with nine genera and negatively with two; acids showed positive correlations with nine genera and negative correlations with six; aldehydes correlated positively with two genera; alcohols demonstrated positive correlations with nine genera and negative correlation with one; while phenolics correlated positively with eight genera. Importantly, the experimental group's fortified Daqu contributed to the dominance of *Lactobacillus*, *Thermoascus*, and *Aspergillus*, which showed strong positive correlations with key flavor compounds (esters, acids, alcohols, and phenolics), confirming their functional importance in flavor formation. By comparison, the dominant genera in the control group showed less pronounced contributions to flavor compound development.

Furthermore, the interactions between microbial communities, such as synergistic or antagonistic relationships, are inferred to significantly influence the formation of flavor compounds. In the experimental group, for instance, synergistic metabolic relationships likely existed among the dominant genera *Lactobacillus*, *Thermoascus*, and *Aspergillus*. *Lactobacillus* may have contributed to a lower environmental pH through acid production, thereby creating favorable conditions for the growth and enzymatic activities of *Thermoascus* and *Aspergillus*. In return, these fungi potentially secreted proteases and amylases, degrading macromolecular substrates into precursors that facilitated the metabolic activities of *Lactobacillus* and other bacteria. This inferred model of cross-kingdom synergy finds strong support in a recent study on Changle sweet rice wine, where the cooperative interaction between Rhizopus and Bacillus was identified as the core mechanism driving the synthesis of characteristic esters and aroma compounds (Zhou et al., 2025). Furthermore, they extend the conceptual framework established by Xu et al. (2025), who demonstrated that multi-starter systems functionally reshape microbial communities to enhance acetate ester and medium-chain fatty acid ethyl ester production. Our study demonstrates that YB13 introduction creates a similarly effective synergistic network in Mixed-flavor Baijiu, specifically involving *Lactobacillus*, *Thermoascus*, and *Aspergillus*. This putative cooperation could have synergistically enhanced the synthesis of key flavor compounds, such as esters, acids, and alcohols. Conversely, the microbial community structure in the control group was relatively simpler and lacked such functional synergies among dominant taxa, potentially leading to incomplete flavor synthesis pathways and a less complex flavor profile. Future studies employing co-culture experiments or metatranscriptomics are warranted to validate these microbial interaction mechanisms.

It should be noted that the correlations between microorganisms and flavor compounds identified in this study are based on Pearson correlation coefficients, and these associations might be influenced by co-variation with key physicochemical parameters (e.g., acidity, starch content) in the fermentation system. Although we provide indirect contextual support for these correlations through the separate analyses of physicochemical parameters versus flavor (Section 3.11) and versus microbial community (Section 3.12), future studies employing more focused experimental designs and larger sample sizes will be necessary to apply advanced statistical models like partial correlation analysis for precisely parsing the direct causal links.

### 3.14 Sensory evaluation of raw liquor

**Fig. 12:**
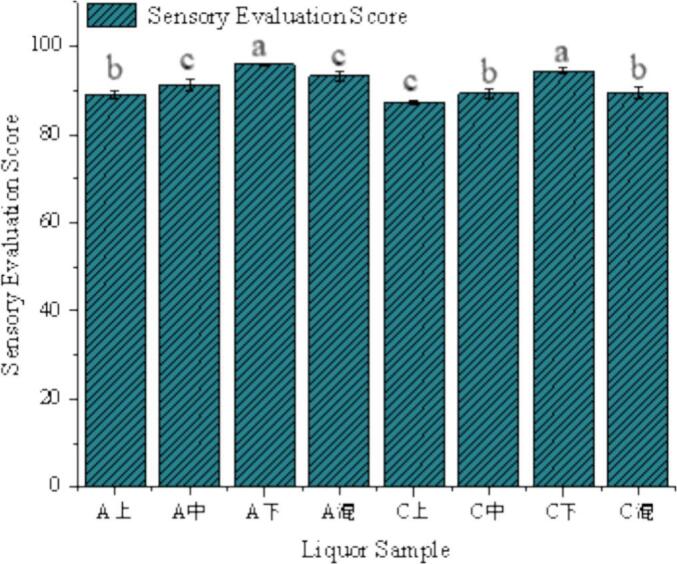
Sensory evaluation of raw baijiu. (A) Experimental group; (B) Control group.

The sensory evaluation results demonstrated significantly higher scores (*p* < 0.05) for the experimental group compared to the control across all sampled layers (upper, middle, lower, and mixed), respectively, with a notable mean difference of 3.63 points between mixed samples (Fig. 12). These findings clearly indicate that the incorporation of Fortified Daqu during fermentation yielded baijiu of markedly superior quality, as evidenced by the comprehensive sensory assessment. The evaluators consistently noted that the experimental group's liquor samples exhibited particularly prominent advantages in cellar aroma intensity, aroma harmony, and taste fullness. These results clearly demonstrate that the addition of fortified Daqu during fermentation significantly enhanced the overall sensory quality of the baijiu. The reliability of these findings is supported by the replicated evaluations conducted by trained panelists. The significant advantage in sensory scores for the experimental group's base liquor corresponds consistently with the higher levels of multiple key flavor substances detected during its fermentation process. In particular, the significantly increased levels of compounds in the experimental group—such as TTMP (contributing nutty aroma), ethyl myristate (contributing sweet aroma and mellow sensation), and acetic acid (providing necessary acidity support) (see Section 3.7)—are considered core contributors collectively enhancing its ‘cellar-rich aroma,’ ‘aroma harmony,’ and ‘taste fullness.’ Future research will conduct systematic flavoromics and quantitative descriptive sensory analysis specifically on the finished liquor to establish more precise quantitative relationship models.

## 4 Discussion

This study demonstrates that applying TTMP-high-producing *B. halotolerans* YB13 in fortified Daqu enhances the Jiangxiang-type Baijiu fermentation process and final product quality. An integrative framework is proposed: YB13 inoculation initially enhanced saccharification capacity and protease activity (Sections 3.3, 3.5), modifying the physicochemical microenvironment. This, in turn, drove microbial community restructuring (Section 3.10), selecting for a functional consortium. The metabolic activities of this consortium subsequently led to significantly increased synthesis of TTMP (Section 3.6) and key flavor compounds (Section 3.7). These molecular changes were correlated with microbial shifts (Sections 3.11–3.13) and were ultimately reflected in significantly improved sensory quality of the base liquor (Section 3.14). The following discussion elaborates on this framework.

The primary bacterial strains responsible for tetramethylpyrazine (TTMP) biosynthesis during baijiu fermentation are members of the *B.* genus. Notably, *B. amyloliquefaciens* has been reported to produce 1.28 mg/g of TTMP, while *B. velezensis* YB26—isolated by Zhuansun et al—demonstrates a higher yield of 4.11 mg/g (Zhuansun., 2023). In this study, strain YB13 was molecularly identified as *B. halotolerans*, representing the first report of TTMP production by this species. Following plasma mutagenesis, the strain achieved a yield of 2.859 mg/g, placing it within the medium-to-high production range among known TTMP-producing *B.* strains.

*B.* species serve as key producers of tetramethylpyrazine (TTMP) during baijiu fermentation. While *B. amyloliquefaciens* yields 1.28 mg/g TTMP and *B. velezensis* YB26 produces 4.11 mg/g, this study identifies *B. halotolerans* YB13 as a novel TTMP-producing strain. After plasma mutagenesis, YB13 achieved a TTMP yield of 2.859 mg/g, placing it in the medium–high range among *B.* strains.

Combining the observed significant increase in TTMP yield by YB13 during solid-state fermentation (Section 3.1) with its promotion of starch degradation and reducing sugar accumulation in the early fermentation stage (Section 3.3), we speculate that its high production trait may be related to its efficient carbon metabolic flux and potential ALS/ALDC enzyme system. This hypothesis awaits verification through future enzyme activity assays or transcriptomic analysis.

In summary, the high TTMP productivity of the mutant strain YB13 stems from synergistic advantages in genetic background, metabolic pathway efficiency, and environmental robustness, making it a promising candidate for application in Mixed-flavor Baijiu fermentation.

The fermentation environment in this study was characterized by high acidity and osmotic pressure. The demonstrated survival and high TTMP synthesis capability of YB13 under these conditions (Section 3.6) provide direct phenotypic evidence of its robust adaptability and metabolic stability within this complex system. Thus, this combination of high TTMP productivity and robust environmental resilience makes *B. halotolerans* a highly promising candidate for application in brewing systems, where stability and performance under stress are critical (Lin et al., 2025). Addionally*, B. halotolerans* contributes significantly to flavor development in baijiu fermentation by producing a diverse range of flavor compounds, such as esters, organic acids, pyrazines, and terpenes (Liu et al., 2022). It also synthesizes key flavor precursors, including acetoin and amino acids, which are essential for the characteristic aroma of baijiu. Furthermore, its acid-resistant α-amylase enhances starch degradation, facilitating the generation of additional flavor-enhancing metabolites (Tu et al., 2022). Beyond flavor formation, *B. halotolerans* improves sensory quality by reducing bitterness and modulating acidity, leading to a more balanced and refined taste profile (Bo et al., 2023).

The differences in physicochemical indices, enzyme activity, flavor compounds, and microbial succession between the control and experimental groups observed in this study are attributed to the addition of enriched koji during brewing process. These variations may result from the interactions between *B. halotolerans* (introduced via the enriched koji), its enzymatic activities, and the surrounding microbial community (Li et al., 2019; Li et al., 2025). These interactions likely promoted changes in physicochemical conditions, enzyme activity profiles, and microbial population dynamics during fermentation. As a result, the experimental group exhibited a more diverse array of flavor compounds and improved baijiu quality. To further elucidate the mechanism behind the enhancement in liquor quality, the interrelationships among these influencing factors and the various indices were analyzed, providing insights into how these perturbations contribute to quality improvement from a correlation-based perspective:

(1) The experimental group inoculated with *B. halotolerans*-enriched koji demonstrated significantly stronger correlations between physicochemical parameters and flavor compounds compared to controls. Starch emerged as the dominant factor, showing significant correlations with 33 flavor compounds, while the control group exhibited only nine significant correlations without a predominant factor. This highlights starch's pivotal role in baijiu fermentation, where its conversion to ethanol simultaneously generates diverse flavor components including acids, alcohols, esters, aldehydes (ketones), and aromatic compounds (Wu et al., 2024). Acid protease played an important role in promoting protein hydrolysis and improving amino acid transformation efficiency during fermentation, thereby supplying essential precursors for the synthesis of flavor compounds. This indicates that even without the addition of exogenous enzymes, the effective utilization of the indigenous microbial community can significantly enhance the quality of the liquor. The enriched koji's enhancement of these starch-driven transformations resulted in greater flavor diversity and superior liquor quality.
(2) During the brewing process of Chinese Baijiu, the experimental group, which was inoculated with a fortified starter culture, showed a close correlation between dominant bacterial genera—such as *Lactobacillus*, *Thermoascus*, and *Aspergillus*—and the formation of various flavor compounds, including esters, acids, and alcohols (Guan et al., 2023; Wu et al., 2023). Through complex metabolic activities, these microorganisms significantly promoted the synthesis of key aroma components such as ethyl hexanoate and ethyl palmitate, exhibiting a strong positive correlation with these compounds. This microbial synergy substantially enhanced the aromatic complexity and taste profile of the final product. In contrast, the control group exhibited lower correlations between flavor compounds (esters, acids, and alcohols) and dominant microbial genera, suggesting a weaker capacity to promote flavor compound formation during fermentation, resulting in a relatively simple and less distinctive flavor profile. Therefore, brewing with a fortified starter culture rich in diverse microbial resources not only increases the content and diversity of flavor compounds but also enriches the overall flavor, leading to a superior sensory profile. This highlights the significant advantages of the experimental group in improving both the quality and flavor characteristics of Chinese Baijiu.

## 5 Conclusion

A salt-tolerant *B. halotolerans* strain with high tetramethylpyrazine (TTMP) production capacity (yielding up to 2.859 mg/g) was isolated for the first time from high-temperature Daqu. When incorporated into enriched koji preparation, this strain significantly enhanced the sensory evaluation scores of the experimental baijiu compared to the control group. This improvement stemmed from multiple factors: the experimental group demonstrated superior physicochemical indices and enzyme activities, with starch and acid protease identified as the primary drivers of flavor compound formation, while starch and α-amylase predominantly influenced microbial community dynamics. In contrast, moisture content played a decisive role in flavor compound generation in the control group, and cellulase activity was the key factor shaping microbial succession. Notably, TTMP levels in both grain mash and fermented grains were markedly higher in the experimental group, confirming the strain's role in elevating TTMP content during fermentation and ultimately improving liquor quality. Furthermore, dominant genera such as *Lactobacillus* and *Aspergillus* in the experimental group exhibited strong correlations with physicochemical parameters, enzyme activities, and key flavor compounds (e.g., esters, acids, and alcohols), whereas the control group's dominant microbes showed weaker associations with these factors. This study successfully demonstrates the application of a TTMP-high-producing *B. halotolerans* YB13 strain in fortified Daqu for Jiangxiang-type Baijiu fermentation. The results indicate that YB13 inoculation optimizes physicochemical parameters and enzyme activity dynamics, restructures the microbial community, and enhances the levels of TTMP and various key flavor compounds, ultimately improving the sensory quality of the base liquor. Consequently, this work lays a solid application foundation for the microbial fortification of Jiangxiang-type Baijiu and provides clear directions for future mechanistic investigations.

Ethical Statement.

This study involving human volunteers for sensory evaluation has obtained ethical approval from Anhui Gold Seed Winery Co., Ltd. After rigorous review by our institutional Ethics Committee, it has been determined that the experimental design and protocol fully adhere to safety and fairness principles. The research poses no harm or risk to participants and is conducted strictly based on the principle of informed consent. The rights and privacy of the participants will be protected to the greatest extent possible. There are no conflicts of interest in either the research content or the outcomes.

## CRediT authorship contribution statement

**Qing Zhao:** Writing – original draft. **Di Wu:** Writing – original draft. **Biao Hao:** Writing – original draft. **Jie Zhang:** Writing – review & editing. **Tianquan Pan:** Writing – original draft. **Jie Qiao:** Writing – original draft. **Huawei Zeng:** Writing – review & editing. **Hongwen Yang:** Writing – review & editing.

## Declaration of competing interest

The authors declare that they have no known competing financial interests or personal relationships that could have appeared to influence the work reported in this paper.

## Data Availability

Data will be made available on request.

## Appendix A Supplementary data

Supplementary material Image 1
