# Supplementary material for: Screening and mutagenesis breeding of B. A salt YB13 for high producing tetramethylpyrazine and the application of its fortified Daqu for improving the quality of mixed-flavor baijiu
Source: Food Chem X. 2025 Dec 11;33:103401. doi: 10.1016/j.fochx.2025.103401 (PMC12765131; doi:10.1016/j.fochx.2025.103401)
Supplement: Supplementary file 1 — Supplementary material [file mmc1.docx]

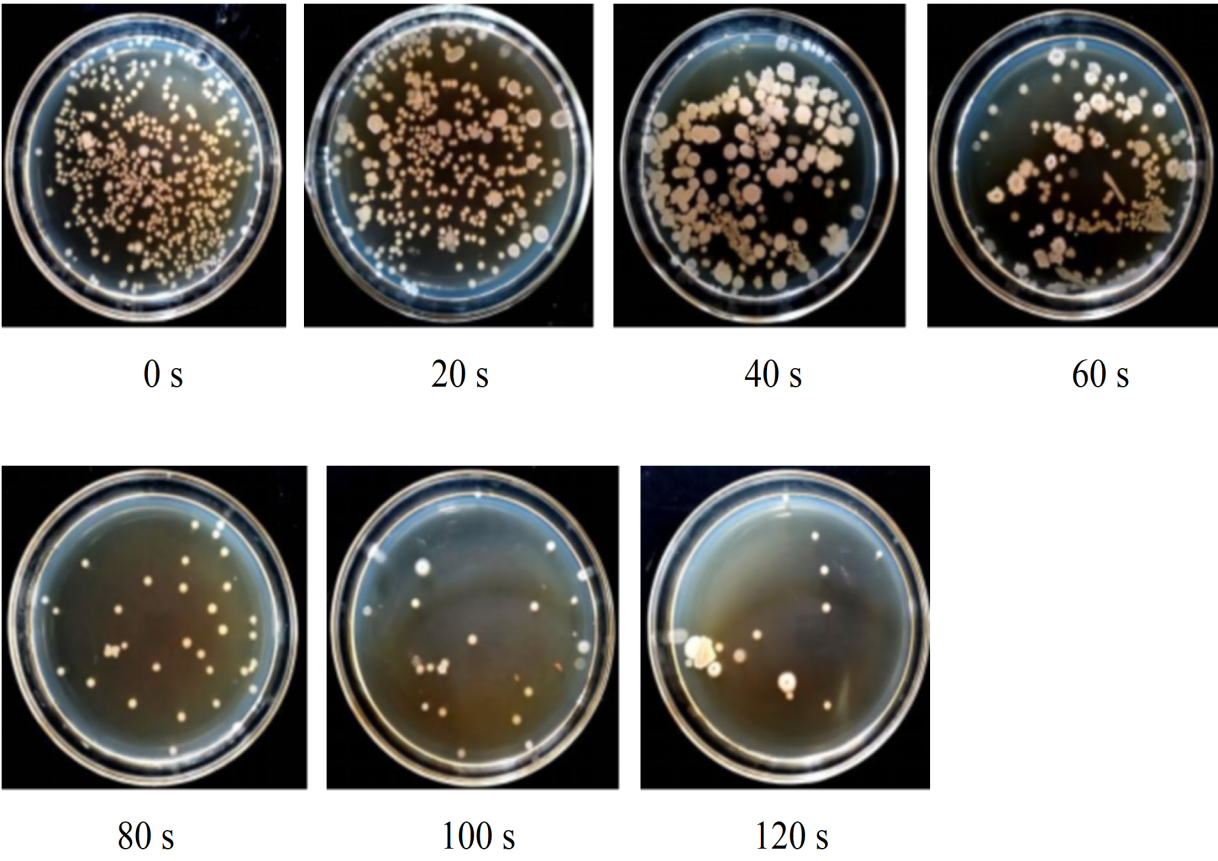


Fig. S-1 The growth status of the strain with dilution ratio of 10^6^ under mutagenesis treatment at varying time intervals


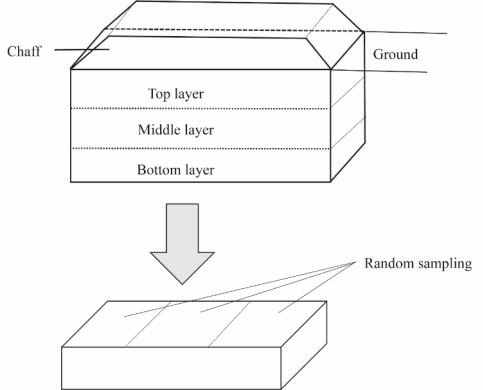


Fig.S-2 Sample Sampling Diagram

Tab. S-1 The comparison of OD value presenting acetoin yield among different strains by using V-P test

| Strain number | OD560 | Strain number | OD560 |
| --- | --- | --- | --- |
| 11-6 | 1.053±0.003 | 4-3 | 0.844±0.012a |
| 1-6 | 0.984±0.006 | 10-1 | 0.716±0.008 |
| 1-5 | 0.640±0.003 | 8-1 | 0.784±0.002 |
| 5-1 | 0.576±0.005 | 6-1 | 0.745±0.008 |
| 11-3 | 0.607±0.006 | 10-2 | 0.536±0.005 |
| 10-4 | 0.591±0.010 | 1-3 | 0.325±0.004 |
| 9-4 | 0.556±0.006 | 6-5 | 0.702±0.015 |
| 2-3 | 0.495±0.005 | 5-3 | 1.080±0.006 |
| 2-2 | 0.631±0.003 | 3-6 | 0.437±0.021 |
| 7-3 | 0.938±0.003 | 4-1 | 0.660±0.026 |
| 7-2 | 0.523±0.003 | 6-3 | 0.498±0.015 |
| 3-5 | 0.323±0.004 | 11-4 | 0.819±0.005 |
| 6-2 | 0.584±0.005 | 8-2 | 0.375±0.003 |
| 8-3 | 0.640±0.003 | 9-3 | 1.141±0.010 |
| 5-2 | 0.694±0.004 | 2-4 | 0.644±0.012 |
| 11-1 | 0.683±0.003 | 1-4 | 0.486±0.005 |
| 9-2 | 0.604±0.014 | 5-4 | 0.459±0.006 |
| 11-5 | 0.427±0.006 | 3-4 | 0.880±0.014 |
| 6-4 | 0.942±0.010 |  |  |

Tab. S-2 The yield of TTMP produced by liquid fermentation of the strain.

| Strain number | TTMP yield（mg/L) | Strain number | TTMP yield（mg/L) |
| --- | --- | --- | --- |
| 7-2 | 15.637±1.000 | 5-1 | 0.320±0.038 |
| 7-1 | 43.045±0.226 | 1-6-1 | 0.157±0.017 |
| 8-3 | 17.936±0.404 | 1-3 | 0.321±0.140 |
| 3-4 | 45.374±1.500 | 11-1 | 0.745±0.078 |
| 7-4 | 13.011±1.153 | 8-1 | 2.305±0.372 |
| 6-3 | 16.760±0.802 | 11-7 | 0.139±0.033 |
| 1-5 | 18.598±0.780 | 11-5 | 8.753±0.673 |
| 8-1 | 0.118±0.076 | 5-3 | 0.106±0.021 |
| 10-1 | 46.513±0.673 | 6-5 | 0.133±0.037 |
| 9-2 | 30.760±0.539 | 8-2 | 0.119±0.015 |
| 5-4 | 30.157±2.373 | 10-4 | 29.085±0.978 |
| 8-3 | 11.424±1.083 | 10-2 | 0.067±0.032 |
| 1-1 | 28.463±1.514 | 7-4 | 0.126±0.010 |
| 11-2 | 29.844±1.300 | 1-4 | 7.470±0.331 |
| 6-5 | 28.170±0.753 | 4-1 | 6.259±0.250 |
| 11-6 | 18.720±0.358 | 11-3 | 6.726±0.227 |
| 6-2 | 0.115±0.010 | 2-4 | 7.564±0.385 |
| 2-4 | 0.168±0.014 | 3-6 | 4.661±0.191 |

Tab.S-3 The yield of TTMP produced by solid-state fermentation of the strains.

| Strain number | TTMP yield（mg/g) | Strain number | TTMP yield（mg/g) |
| --- | --- | --- | --- |
| 6-5 | 0.406±0.006 | 11-2 | 0.068±0.007 |
| 1-6-2 | 0.049±0.004 | 5-4 | 0.107±0.013 |
| 1-1 | 0.031±0.005 | 9-2 | 0.021±0.004 |
| 10-1 | 0.024±0.004 | 3-4 | 0.032±0.005 |
| 11-4 | 0.030±0.005 | 11-3 | 0.026±0.002 |
| 11-6 | 1.366±0.041 | 11-1 | 0.034±0.003 |
| 1-6-1 | 0.179±0.007 | 3-6 | 0.050±0.004 |
| 7-2 | 0.032±0.005 | 8-3 | 0.113±0.019 |
| 7-1 | 0.396±0.014 | 11-5 | 0.889±0.006 |

Tab.S-4 The production of TTMP content by solid-state fermentation of the mutated strains

| Strain number | TTMP yield（mg/g) | Strain number | TTMP yield（mg/g) |
| --- | --- | --- | --- |
| YB1 | 2.084±0.032 | YB22 | 1.762±0.018 |
| YB4 | 2.254±0.018 | YB27 | 1.952±0.007 |
| YB5 | 1.785±0.019 | YB28 | 2.125±0.023 |
| YB8 | 2.428±0.012 | YB29 | 2.236±0.039 |
| YB9 | 2.247±0.000 | YB31 | 2.236±0.056 |
| YB13 | 2.859±0.017 | YB32 | 2.149±0.054 |
| YB14 | 2.220±0.008 | YB35 | 1.666±0.040 |
| YB17 | 2.310±0.027 | YB36 | 1.809±0.002 |

Tab S-5 Dynamic changes of flavor compound content during the fermentation process of liquor cultures in the experimental group

| **组分** | | **Concentration（mg/Kg)** | | | | | | | |
| --- | --- | --- | --- | --- | --- | --- | --- | --- | --- |
|  |  | **A1** | **A4** | **A7** | **A12** | **A17** | **A27** | **A42** | **A62** |
| butyrolactone | E1 | 0.56±0.03 | 1.05±0.31 | 0.35±0.36 | 0.57±0.50 | 0.90±0.78 | 0.81±0.70 | 1.36±0.41 | 2.75±0.38 |
| dibutyl phthalate | E2 | 1.27±0.03 | 1.08±0.49 | 0.59±0.11 | 0.42±0.28 | 0.72±0.40 | 0.35±0.11 | 0.71±015 | 0.83±0.28 |
| Diethyl glutarate | E3 | 2.04±0.03 | 3.27±0.89 | 1.60±0.14 | 2.31±0.49 | 3.13±0.39 | 2.62±0.40 | 3.71±0.91 | 9.89±0.82 |
| Isocyanates | E4 | 14.78±0.60 | 31.54±4.14 | 0.36±0.31 | ND | ND | ND | 1.17±0.88 | 1.91±0.25 |
| ethyl benzoate | E5 | ND | ND | 0.96±0.04 | 1.34±0.67 | 1.88±0.08 | 1.22±0.08 | 1.66±0.56 | 3.09±0.70 |
| isobutyl salicylate | E6 | ND | ND | 1.11±0.14 | 1.95±0.45 | 3.50±0.62 | 2.63±0.17 | 3.77±0.02 | 7.37±0.44 |
| Caprolactone | E7 | ND | 0.74±0.16 | 0.26±0.24 | 0.46±0.08 | 0.73±0.03 | ND | 0.58±0.10 | 0.70±0.06 |
| Ethyl hexadecanoate | E8 | 40.83±4.39 | 131.40±49.45 | 49.03±10.06 | 103.05±8.34 | 182.99±1.74 | 166.17±18.19 | 130.11±18.97 | 225.16±34.72 |
| Diethyl azelaite | E9 | 2.69±0.57 | 2.36±0.18 | 1.87±0.34 | 1.44±0.71 | 2.81±0.60 | 2.43±0.85 | 2.77±0.11 | 8.49±0.54 |
| Hexyl Hexanoate | E10 | ND | 0.87±0.34 | ND | 0.67±0.05 | 0.99±0.08 | 0.89±0.08 | ND | ND |
| furfuryl acetate | E11 | 3.39±0.15 | 4.46±0.35 | 1.69±0.47 | 3.84±0.54 | 4.35±0.82 | 3.46±0.88 | ND | 11.24±1.88 |
| 1,2-propanediol, 1-acetate | E12 | ND | ND | 1.13±0.04 | 2.88±0.28 | 7.12±0.92 | 6.15±0.76 | 9.50±0.93 | 13.50±2.20 |
| propyl lactate | E13 | 0.27±0.04 | ND | 2.05±0.44 | 2.01±0.08 | 3.11±0.26 | 4.40±0.63 | 6.70±0.20 | 20.83±5.98 |
| 9-Hexadecenoic acid ethyl ester | E14 | 2.58±0.36 | 6.48±0.93 | 2.24±089 | 0.80±0.42 | 9.88±0.84 | 9.38±1.77 | 6.90±0.75 | 16.02±2.46 |
| ethyl linoleate | E15 | 9.26±0.68 | 29.94±4.74 | 6.52±0.98 | 21.25±5.66 | 45.26±2.90 | 31.51±4.42 | 25.36±1.96 | 78.11±4.24 |
| Ethyl 2-furancarboxylate | E16 | ND | ND | 0.17±0.1 | 0.42±0.07 | 0.48±0.02 | 0.73±0.08 | 0.93±0.2 | 2.36±0.02 |
| ethyl valerate | E17 | 14.57±0.30 | 22.58±4.80 | 10.30±130 | 9.37±3.86 | 15.00±3.19 | 6.35±1.64 | 7.09±0.23 | 19.27±6.52 |
| Ethyl pentadecanoate | E18 | 0.71±020 | 2.49±0.85 | 0.70±0.30 | 1.96±1.80 | 3.26±0.34 | 3.21±0.79 | 2.52±0.53 | 5.97±0.20 |
| N-acetyl-L-leucine ethyl ester | E19 | 0.28±0.07 | 0.58±0.30 | ND | 0.47±0.07 | 0.69±0.03 | 0.52±0.09 | 0.88±0.06 | 1.98±0.51 |
| Diethyl phthalate | E20 | 1.74±0.23 | 1.62±0.86 | 1.47±0.29 | 0.97±0.37 | 1.77±0.21 | 1.33±0.41 | 1.50±0.56 | 5.20±0.11 |
| Diethyl pimelate | E21 | 0.78±0.01 | 0.93±0.03 | 0.58±0.07 | 0.52±0.14 | 0.85±0.04 | 0.64±0.17 | 0.82±0.03 | 2.41±0.31 |
| Ethyl 3-phenylpropionate | E22 | 16.64±0.19 | 31.09±8.48 | 17.60±1.11 | 16.72±0.3 | 25.64±1.78 | 23.35±4.78 | 20.92±0.69 | 66.14±18.92 |
| butyl salicylate | E23 | 9.56±1.50 | 20.58±7.69 | 12.54±1.43 | 23.29±4.74 | 45.94±5.33 | 34.15±1.20 | 45.78±5.40 | 128.88±13.54 |
| 3-methylthiopropionic acid ethyl ester | E24 | 4.12±0.22 | 7.71±3.82 | ND | 4.91±0.53 | 9.18±0.24 | 7.01±0.01 | 11.28±0.94 | 26.11±0.96 |
| Dimethyl phthalate | E25 | 0.29±0.00 | 0.47±0.20 | 0.35±0.09 | 0.38±0.06 | 0.78±0.20 | 0.43±0.02 | 0.82±0.33 | 1.70±0.71 |
| benzyl acetate | E26 | ND | ND | ND | 2.21±0.49 | 1.63±0.41 | 1.90±0.39 | 2.10±0.61 | 5.66±0.56 |
| butyl lactate | E27 | ND | 1.20±0.20 | 0.80±0.16 | 1.56±0.16 | 3.58±076 | 1.33±0.08 | 2.32±0.16 | 12.61±1.26 |
| Ethyl tetradecanoate | E28 | 3.75±0.62 | 10.92±3.31 | 3.51±0.23 | 7.66±0.94 | 13.83±1.82 | 13.65±3.24 | 11.91±0.38 | 23.16±1.78 |
| butyl acetate | E29 | 9.50±0.77 | 4.51±0.21 | 6.72±0.99 | 6.25±0.84 | 10.21±0.18 | 4.61±0.32 | 4.13±0.41 | 10.47±0.09 |
| ethyl caproate | E30 | 75.15±4.64 | 129.85±7.99 | 72.22±5.47 | 60.67±13.95 | 200.67±62.31 | 114.50±16.22 | 79.74±3.98 | 183.00±36.92 |
| diethyl succinate | E31 | 25.51±0.59 | 48.18±11.29 | 20.05±2.10 | 31.89±5.83 | 45.16±5.53 | 38.96±5.73 | 56.18±11.88 | 125.60±8.69 |
| Octyl salicylate | E32 | 0.46±0.02 | 1.03±0.74 | 0.66±0.06 | 0.44±0.04 | 1.44±0.26 | 0.60±0.02 | 0.69±0.06 |  |
| ethyl oleate | E33 | 13.69±4.01 | 35.31±5.11 | 13.43±3.48 | 2.90±0.04 | 48.37±0.64 | 38.36±1.96 | 32.01±2.97 | 91.33±41.89 |
| Ethyl Stearate | E34 | 0.63±0.03 | 1.90±0.09 | 0.84±0.33 | 1.34±0.05 | 2.31±0.04 | 1.74±0.11 | 1.28±0.04 | 4.02±0.02 |
| ethyl decanoate | E35 | ND | 4.41±1.15 | 1.13±0.45 | 2.86±0.34 | 6.17±0.20 | 3.63±0.67 | 3.11±0.34 | 4.03±0.87 |
| Ethyl octanoate | E36 | 1.89±0.14 | 4.24±0.12 | 2.30±0.31 | 3.50±0.63 | 7.72±0.73 | 3.11±0.31 | ND | 16.70±1.08 |
| ethyl enanthate | E37 | 14.68±0.51 | 17.89±0.53 | 14.06±168 | 8.00±0.58 | 15.35±0.52 | 9.99±2.38 | 8.88±0.93 | 30.03±5.47 |
| Ethyl Butyrate | E38 | 14.42±2.18 | 15.58±3.69 | 11.89±2.35 | 15.33±2.62 | 13.33±1.56 | 13.90±0.43 | 17.74±2.00 | 36.67±3.66 |
| 2-phenylethyl dicarbonate | E39 | 0.20±0.21 | 0.45±0.04 | ND | 0.04±0.05 | ND | ND | 0.07±0.03 | ND |
| C-nonanolactone | E40 | 4.54±0.09 | 7.52±0.28 | 5.73±0.4 | 8.18±1.55 | 14.62±1.48 | 9.86±1.02 | 11.63±1.73 | 28.31±4.41 |
| 2-Hydroxy-4-methylpentanoic acid ethyl ester | E41 | 14.11±1.14 | 26.65±5.72 | 30.10±3.63 | 55.00±7.80 | 90.20±6.77 | 79.74±9.74 | 105.82±22.99 | 254.94±12.58 |
| Phenylethyl Acetate | E42 | 43.94±1.24 | 58.08±3.37 | 43.75±3.03 | 52.26±6.23 | 93.34±5.23 | 77.10±13.61 | 83.35±25.46 | ND |
| Ethyl phenylacetate | E43 | 17.10±0.07 | 32.32±6.47 | 22.31±1.59 | 29.93±1.72 | 50.31±151 | 52.15±9.34 | 63.83±2.49 | ND |
| Ethyl thioacetate | E44 | 0.84±0.09 | 1.24±0.37 | 1.08±0.08 | 1.51±0.22 | ND | 1.94±0.39 | 2.98±0.81 | 11.19±1.73 |
| Hept-4-ylisobutyl phthalate | E45 | 0.87±0.13 | ND | 0.42±0.09 | 0.25±0.02 | 0.45±0.04 | 0.25±0.02 | ND | 0.50±0.04 |
| 3-methylbutyl methoxyacetate | E46 | ND | ND | ND | 27.98±2.73 | ND | 52.01±7.14 | 64.19±3.74 | 111.15±9.90 |
| 1,2-propanediol, 2-acetate | E47 | ND | ND | ND | 0.51±0.04 | 2.78±0.20 | 2.63±0.35 | 4.39±075 | 5.59±1.05 |
| 2-methylpropionic acid | E48 | 17.30±1.36 | 13.81±0.14 | 9.14±2.51 | ND | 21.60±6.13 | 11.46±3.12 | ND | 18.21±1.77 |
| 4-methylvaleric acid | E49 | 2.26±0.37 | 3.91±0.08 | 1.23±0.13 | 1.54±0.31 | 2.63±0.51 | 1.69±0.34 | 1.93±0.56 | 6.72±1.08 |
| acetic acid | E50 | 67.37±12.99 | 128.11±23.69 | 115.08±33.21 | 149.57±25.24 | 450.60±9.10 | 251.17±33.66 | 328.71±16.36 | 375.12±17.53 |
| 3-Methylbutanoic acid | E51 | 69.63±5.26 | 66.99±6.54 | 45.58±6.86 | 53.72±8.65 | 96.69±7.29 | 67.76±5.86 | 73.02±11.44 | 120.98±22.78 |
| hexanoic acid | E52 | 105.25±18.69 | 247.86±17.66 | 83.11±10.53 | 115.51±19.96 | 215.02±26.23 | 146.88±11.97 | 204.45±19.65 | ND |
| heptanoic acid | E53 | 11.73±3.93 | 17.90±0.82 | 6.30±0.90 | 7.37±0.93 | 15.13±5.38 | 7.88±0.2 | 18.85±7.10 | 41.06±2.97 |
| Valproic acid | E54 | 17.45±2.43 | 44.02±1.76 | 9.76±1.15 | 12.82±1.84 | 23.35±3.33 | 15.39±1.82 | 16.20±2.68 | 50.73±1.79 |
| butyrate | E55 | 24.35±0.97 | 46.00±8.65 | 13.34±2.12 | 16.48±2.10 | 27.94±444 | 18.06±2.27 | 19.08±4.00 | 68.49±1.32 |
| Benzoic acid | E56 | ND | ND | 3.03±0.71 | 6.34±0.29 | 11.56±3.38 | 8.39±274 | 9.04±2.50 | 28.96±4.22 |
| 2-Hydroxy-2-methylpropyl propionate | E57 | ND | 2.05±0.31 | 2.59±0.28 | 4.01±0.52 | 6.35±0.86 | 5.27±0.84 | 8.33±0.82 | 19.62±2.06 |
| decanoic acid | E58 | ND | ND | 2.40±0.55 | 2.42±0.91 | 4.04±0.23 | 1.78±0.84 | 2.36±0.79 | 8.43±2.28 |
| bitter | E59 | ND | ND | 10.64±1.98 | 10.85±3.93 | 24.60±9.05 | 13.97±1.79 | 44.53±9.85 | 71.75±2.47 |
| Nonanoic acid | E60 | ND | ND | 5.89±0.46 | 3.77±0.04 | 7.58±0.46 | 4.20±0.95 | 3.40±0.00 | 13.34±1.60 |
| Phenylacetic acid | E61 | ND | ND | 2.07±0.89 | 6.02±0.73 | 11.04±0.96 | 7.27±0.84 | 8.80±010 | 23.43±0.17 |
| 3-Furoxaldehyde | E62 | 2.67± | ND | 0.86±0.14 | 1.32±0.12 | 2.45±0.37 | 2.09±0.34 | 2.67±0.35 | 4.98±00.58 |
| phenylacetaldehyde | E63 | 5.51±0.75 | ND | 5.00±0.65 | 7.09±0.48 | 11.79±1.62 | 9.43±1.75 | 10.59±348 | 31.89±3.89 |
| N-methyl-2-pyrroleformaldehyde | E64 | 0.25±0.02 | 0.67±0.20 | 0.11±0.02 | 0.25±0.05 | 0.25±0.02 | 0.24±0.01 | 0.39±0.06 | 0.72±0.03 |
| benzaldehyde | E65 | 8.60±0.16 | 10.94±275 | 8.61±1.44 | 18.07±4.36 | 29.25±2.86 | 26.29±3.91 | 21.75±5.34 | 110.37±27.73 |
| 3,4-Dimethylbenzaldehyde | E66 | ND | ND | 2.01±0.89 | 5.52±0.42 | 17.58±4.79 | ND | 13.16±1.29 | 15.39±1.88 |
| 3-hydroxy-4-methoxybenzaldehyde | E67 | ND | 6.05±0.71 | 3.45±0.16 | 3.58±3.10 | 8.15±0.14 | 5.41±0.85 | 8.03±1.48 | ND |
| 3-methylthiopropanol | E68 | 1.57±0.05 | 2.41±0.67 | 1.47±0.12 | 1.82±0.38 | 2.53±0.31 | 1.73±0.34 | 2.23±0.49 | 4.92±0.42 |
| β - Ethylphenylethanol | E69 | 1.52±0.05 | 1.73±0.36 | 1.78±0.06 | 1.20±0.08 | 1.34±0.16 | 2.32±0.56 | 2.24±0.19 | 5.06±0.07 |
| 1-Nonanol | E70 | ND | 26.92±5.96 | 7.45±0.61 | 13.01±1.62 | ND | 26.74±2.62 | 34.95±572 | ND |
| 2-Furamol | E71 | 0.45±0.01 | 0.58±0.18 | 0.33±0.06 | 0.47±0.10 | 0.78±0.10 | 0.55±0.07 | ND | 1.26±0.22 |
| 3-Phenylpropanol | E72 | 0.32±0.02 | 0.47±0.13 | 0.29±0.02 | 0.40±0.09 | 0.73±0.10 | 0.51±0.07 | 0.58±0.11 | ND |
| 2-phenyl-1-propanol | E73 | 9.80±0.04 | 12.86±1.61 | 8.90±0.96 | 14.19±1.91 | 21.40±3.33 | 15.61±1.87 | 19.01±2.35 | ND |
| Hexanol | E74 | 1.11±0.13 | 1.26±0.22 | 0.58±0.07 | 0.58±0.24 | 1.06±0.05 | 0.84±0.1 | 0.87±0.20 | ND |
| 2-ethylhexanol | E75 | 7.00±0.24 | ND | 6.25±0.81 | 9.67±1.61 | 17.47±1.93 | 12.22±123 | 13.81±2.58 | 29.98±3.48 |
| BENZYL ALCOHOL | E76 | ND | 3.99±0.05 | 3.17±0.25 | 3.98±0.82 | 6.58±0.76 | 3.23±0.85 | 5.32±1.06 | 7.84±0.88 |
| 4-phenyl-3-buten-2-ol | E77 | ND | 7.03±0.87 | 13.08±0.80 | 12.62±3.78 | 30.21±9.89 | 16.72±0.88 | 18.05±2.83 | 16.65±3.27 |
| 3-methylacetic acid 1-butanol | E78 | ND | 3.06±0.34 | 2.43±0.24 | 2.14±0.12 | 3.31±0.08 | 2.84±0.97 | 4.22±0.17 | 8.74±00.66 |
| 1-Octanol | E79 | ND | ND | 0.43±0.39 | 1.55±0.32 | 3.10±0.18 | 2.85±0.33 | 3.72±0.11 | ND |
| Furamol 2Me derivatives | E80 | 1.66±0.34 | 1.36±0.01 | 1.20±0.08 | 0.78±0.04 | 0.82±0.73 | 1.35±0.20 | ND | 0.89±0.84 |
| 2,4-di-tert-butylphenol | E81 | 12.47±1.93 | ND | 6.80±0.34 | 10.26±2.07 | 12.85±1.89 | 8.42±1.77 | 14.47±1.60 | 7.19±1.02 |
| 4-vinyl-2-methoxyphenol | E82 | 5.01±0.37 | 7.10±0.94 | 14.05±0.43 | 20.18±4.76 | 38.27±3.63 | 25.95±3.56 | 23.31±5.95 | 87.74±8.38 |
| 4-Ethyl-2-methoxyphenol | E83 | 2.51±0.09 | 3.08±0.82 | 6.96±0.74 | 9.49±0.05 | 25.72±2.96 | 15.39±2.17 | 12.22±3.23 | 43.00±5.38 |
| 4-Ethylphenol | E84 | 7.26±0.56 | 7.09±1.77 | 22.89±2.17 | 42.06±012 | 75.02±8.94 | 46.67±5.59 | 45.39±1.72 | 134.50±6.86 |
| 2-methoxy-5-methylphenol | E85 | 2.13±0.07 | 3.70±0.91 | 1.73±0.22 | 2.41±0.42 | 4.29±0.53 | 2.90±0.33 | 3.28±0.55 | 7.27±0.43 |
| phenol | E86 | 6.75±0.38 | 15.34±3.04 | 5.36±0.56 | 6.88±1.12 | 0.48±0.06 | 8.91±1.19 | 9.39±1.88 | 24.44±3.44 |
| p-cresol | E87 | 5.94±0.43 | 5.94±1.40 | 4.66±0.50 | 6.25±1.06 | 11.69±1.61 | 8.11±1.14 | 9.23±1.67 | 18.89±1.06 |
| 2-methoxyphenol | E88 | 1.57± | 2.41± | 1.47± | 1.82± | 2.53± | 1.73± | 2.23± | 4.92± |
| Pentadecane | E89 | 3.21± | 1.82± | 1.37± | 1.14± | 1.26± | 1.48± |  |  |
| Octamethylcyclopentasiloxane | E90 | 1.05±0.27 | ND | 0.55±0.17 | 0.41±0.23 | 0.79±0.16 | 0.72±0.07 | 0.69±0.39 | 4.40±0.30 |
| Hexamethylcyclooctasiloxane | E91 | 5.44±0.29 | 4.99±0.99 | 2.74±0.73 | 2.77±0.09 | 5.18±0.26 | 5.96±1.54 | 5.38±0.85 | 15.85±0.3 |
| octamethylcyclotetrasiloxane | E92 | 10.53±107 | ND | 9.38±1.35 | ND | 0.79±0.06 | 8.36±1.45 | ND | 101.23±6.39 |
| hexamethylcyclotrisiloxane | E93 | 0.49±0.16 | 0.43±0.15 | 0.16±0.01 | ND | 0.89±0.12 | 0.30±0.02 | ND | 20.73±2.48 |
| decamethylcyclopentasiloxane | E94 | 5.18±0.24 | 6.87±1.39 | 3.53±0.12 | 4.42±0.60 | 12.50±0.25 | 8.47±0.49 | 5.57±0.90 | 49.83±4.48 |
| Dodecarboxyl cyclohexyl siloxane | E95 | 25.56±4.81 | 20.87±0.58 | 18.40±0.07 | ND | 35.29±6.00 | 32.58±1.39 | 31.25±1.68 | 42.77±1.16 |
| Tetradecyl cycloheptylsiloxane | E96 | 30.88±5.07 | 34.64±2.99 | 18.34±1.14 | 18.83±1.66 | 36.35±115 | 49.94±2.71 | 45.67±3.86 | 38.58±1.11 |
| 3- (1-methylethyl) oxetane | E97 | ND | ND | 42.47±3.55 | 70.77±6.60 | ND | 85.70±767 | 133.38±21.73 | 136.46±18.18 |
| Trimethylpyrazine | E98 | 1.08±0.49 | 0.99±0.2 | 0.38±0.13 | ND | 0.65±0.05 | 0.15±0.01 | 0.83±0.01 | 3.80±0.02 |
| Tetramethylpyrazine | E99 | 1.25±0.09 | 2.03±0.06 | 0.42±0.05 | 0.54±0.21 | ND | 0.16±0.05 | 0.52±0.13 | 0.17±0.03 |

Tab S-6 Dynamic changes of flavor compound content in the fermentation process of control group for wine cultivation

| **组分** | | **Concentration（mg/Kg)** | | | | | | | | |
| --- | --- | --- | --- | --- | --- | --- | --- | --- | --- | --- |
|  |  | **C1** | **C4** | **C7** | **C12** | **C17** | **C27** | **C42** | | **C62** |
| ethyl benzoate | F1 | 1.01±0.89 | 4.47±0.63 | 5.93±0.81 | 2.97±0.56 | 4.55±0.53 | 2.18±0.91 | 0.92±0.43 | 2.58±0.26 | |
| dibutyl phthalate | F2 | 0.52±0.15 | 1.06±0.02 | 1.57±0.72 | 1.07±0.19 | 1.25±0.25 | 0.43±0.16 | 0.56±0.45 | 0.81±0.28 | |
| isobutyl salicylate | F3 | 1.13±0.26 | 4.29±0.13 | 4.05±0.54 | 1.71±0.41 | 1.28±0.11 | 1.31±0.15 | 0.47±0.41 | | 6.32±0.95 |
| Diethyl glutarate | F4 | 2.89±0.47 | 3.43±0.56 | 5.04±0.25 | 1.88±0.46 | 2.23±0.37 | 2.17±0.14 | 1.88±0.71 | | 8.50±0.59 |
| Isocyanates | F5 | ND | 63.14±3.28 | 51.37±8.71 | 0.54±0.13 | 0.88±0.39 | 0.71±0.62 | 19.88±3.98 | | 0.80±0.05 |
| Ethyl hexadecanoate | F6 | 50.36±0.63 | 283.36±15.01 | 387.03±8.92 | 91.81±6.40 | 92.64±5.93 | 66.37±4.13 | 80.06±6.45 | | 268.68±10.50 |
| Diethyl azelaite | F7 | 3.33±0.90 | 6.72±0.24 | 11.48±0.98 | 3.93±0.22 | 5.24±0.88 | 2.53±0.40 | 1.47±0.89 | | 15.21±0.31 |
| 2-furanmethanol acetate | F8 | 1.33±0.15 | 3.47±0.22 | 3.93±0.87 | 1.68±0.50 | 1.98±0.74 | 2.29±0.10 | 3.05±0.72 | | 4.08±0.54 |
| Ethyl 2-oxopropionate | F9 | ND | 4.51±0.44 | 4.42±0.01 | 1.22±0.15 | 1.02±0.35 | ND | 0.61±0.36 | | ND |
| 2-Hydroxypropyl Propionate | F10 | 0.36±0.15 | 4.62±0.73 | 6.19±0.72 | 1.86±0.27 | 1.49±0.30 | 1.06±0.93 | ND | | ND |
| 9-Hexadecenoic acid ethyl ester | F11 | 3.45±0.90 | 15.80±1.03 | 22.42±0.75 | 5.12±0.01 | 4.43±0.99 | 3.45±0.99 | 2.49±0.94 | | 18.20±0.13 |
| ethyl linoleate | F12 | 12.48±3.71 | 58.78±4.57 | 82.45±5.18 | 20.85±5.89 | 20.41±0.61 | 15.04±1.31 | 22.07±3.50 | | 88.19±4.65 |
| Ethyl 4-oxovalerate | F13 | 1.71±0.67 | 1.66±0.45 | 2.31±0.49 | 0.64±0.16 | 3.35±0.11 | 1.36±0.19 | 1.01±0.35 | | 5.48±0.95 |
| ethyl valerate | F14 | 25.12±1.34 | ND | 7.27±1.02 | 3.16±0.57 | ND | 6.65±1.83 | 6.96±1.45 | | 79.04±7.67 |
| Ethyl pentadecanoate | F15 | 0.57±0.51 | 5.48±0.31 | 7.19±0.13 | 1.58±0.39 | 1.72±0.59 | 0.99±0.86 | 1.51±0.67 | | 5.95±0.26 |
| Diethyl phthalate | F16 | 2.19±0.28 | 3.54±0.01 | 6.61±0.87 | 2.64±0.22 | 2.83±0.92 | 1.56±0.21 | 1.35±0.78 | | 6.30±0.41 |
| Diethyl pimelate | F17 | 0.99±0.14 | 1.10±0.10 | 1.89±0.18 | 0.74±0.13 | 0.75±0.34 | 0.60±0.07 | 0.59±0.27 | | 3.13±0.51 |
| Ethyl phenylpropionate | F18 | 20.39±0.92 | 19.99±2.40 | 29.07±2.72 | 16.86±3.06 | 16.48±0.11 | 10.83±0.86 | 9.96±0.30 | | 82.56±3.53 |
| isoamyl lactate | F19 | ND | 94.61±16.02 | 131.13±5.27 | ND | 31.93±2.59 | 26.77±3.37 | ND | | 68.95±6.95 |
| 2-Hydroxy-3-phenylpropanoic acid ethyl ester | F20 | 17.30±2.52 | 77.37±2.52 | 72.00±5.40 | 24.21±0.41 | 33.94±0.20 | 27.51±4.20 | 22.23±0.33 | | 153.08±13.71 |
| ethyl acetate | F21 | 72.72±7.49 | ND | ND | 209.55±4.80 | 158.63±6.51 | 213.19±9.19 | 94.60±20.52 | | 118.80±6.81 |
| butyl lactate | F22 | 1.01±0.33 | 3.20±0.24 | 6.39±0.36 | 1.66±0.35 | 1.80±0.22 | 1.73±0.32 | 0.56±0.30 | | 24.48±0.60 |
| Ethyl 3- (methylthio) propionate | F23 | 7.83±1.86 | ND | ND | 5.54±0.69 | 4.92±0.71 | 4.98±0.89 | 2.88±0.95 | | 18.32±0.35 |
| Dimethyl Phthalate | F24 | 0.61±0.16 | 1.67±0.82 | 1.71±0.58 | 0.29±0.09 | 0.33±0.06 | 0.43±0.22 | 0.36±0.24 | | 1.49±0.39 |
| Ethyl Myristate | F25 | 3.77±0.52 | 10.91±2.34 | 12.94±0.57 | 7.13±0.94 | 8.60±0.04 | 5.25±0.55 | 6.05±0.42 | | 20.08±1.37 |
| butyl acetate | F26 | 2.57±0.10 | 7.23±0.37 | 5.65±0.88 | 3.92±0.41 | 3.82±0.34 | 3.14±0.72 | 2.89±0.96 | | 6.08±0.58 |
| ethyl caproate | F27 | 297.25±24.96 | 49.31±3.62 | 88.23±1.95 | 76.47±1.91 | 65.98±0.93 | 64.92±4.53 | 42.13±0.89 | | 346.71±5.17 |
| diethyl succinate | F28 | 41.84±7.68 | 55.30±8.74 | 77.93±1.10 | 32.11±0.49 | 37.59±5.52 | 36.23±2.33 | 36.70±2.66 | | 158.78±23.27 |
| ethyl linolenate | F29 | ND | 1.84±0.09 | 2.82±0.22 | 0.65±0.24 | ND | 0.35±0.30 | 0.55±0.04 | | 2.91±0.44 |
| ethyl oleate | F30 | 22.95±0.63 | 72.12±6.49 | 98.80±8.28 | 26.35±0.85 | 28.59±5.99 | 20.39±1.80 | 27.32±1.22 | | 97.63±3.87 |
| Ethyl octadecanoate | F31 | 1.12±0.51 | 3.86±0.11 | 5.45±0.36 | 1.35±0.38 | 1.50±0.36 | 1.03±0.72 | 1.24±0.15 | | 4.51±0.29 |
| ethyl decanoate | F32 | ND | 7.22±0.23 | 5.31±0.89 | 2.09±0.93 | 3.36±0.69 | 3.94±0.50 | 2.07±0.98 | | 11.02±0.64 |
| Ethyl octanoate | F33 | 7.54±0.98 | ND | 6.84±0.80 | 7.18±0.37 | 6.87±0.26 | 8.48±0.69 | 6.27±0.63 | | 12.58±0.39 |
| ethyl enanthate | F34 | 26.89±7.33 | 12.38±0.36 | 14.86±0.91 | 15.01±0.30 | 10.31±0.34 | 10.84±0.33 | 7.02±0.17 | | 14.07±0.32 |
| Ethyl Butyrate | F35 | 20.32±3.53 | ND | 8.45±0.37 | 12.18±1.05 | 12.56±0.97 | 16.07±2.51 | 8.73±0.95 | | 176.79±3.14 |
| C-nonanolactone | F36 | 6.93±0.95 | 15.91±1.90 | 23.18±0.58 | 6.88±1.71 | 8.14±1.44 | 7.21±0.60 | 6.84±0.55 | | 21.05±0.15 |
| 2-Hydroxy-4-methylpentanoic acid ethyl ester | F37 | 18.07±3.93 | 181.59±23.96 | 178.63±15.43 | 61.49±12.97 | 86.32±11.82 | 79.00±5.88 | 22.15±6.64 | | 195.04±25.70 |
| Phenylethyl Acetate | F38 | 31.59±7.84 | 62.67±9.54 | 86.35±7.79 | 40.20±0.28 | 40.96±7.69 | 34.39±4.63 | 29.72±1.19 | | 83.58±6.31 |
| Ethyl phenylacetate | F39 | 17.57±5.64 | 40.91±5.89 | 57.40±6.69 | 31.63±5.91 | 38.81±5.74 | 36.47±6.57 | 19.09±6.84 | | 44.55±6.69 |
| 3- (Methylthio) acetate propyl ester | F40 | 0.78±0.07 | 1.60±0.27 | 2.26±0.25 | 0.76±0.13 | 0.92±0.16 | ND | 0.48±0.13 | | 2.13±0.19 |
| Hept-4-ylisobutyl phthalate | F41 | 0.24±0.01 | 0.65±0.10 | 0.70±0.30 | 0.79±0.16 | 0.70±0.22 | 0.29±0.02 | 0.30±0.03 | | 0.34±0.03 |
| 9-Tetradecenoic acid ethyl ester | F42 | ND | 2.02±0.33 | 3.60±0.13 | 0.73±0.21 | 0.94±0.28 | 0.48±0.04 | 0.40±0.17 | | 2.59±0.01 |
| 1,2-propanediol, 2-acetate | F43 | 0.57±0.14 | 0.76±0.12 | 0.47±0.04 | 0.19±0.17 | ND | ND | ND | | 2.26±0.63 |
| Ethyl 2-hydroxy-3-methylbutyrate | F44 | ND | 8.26±0.29 | 3.49±0.03 | 1.73±0.02 | ND | 2.60±0.02 | ND | | 3.58±0.03 |
| 2-methylpropionic acid | F45 | 21.72±5.01 | 12.84±1.91 | 13.75±0.78 | 3.94±0.77 | 4.27±0.59 | 3.27±0.05 | 5.37±0.16 | | 16.99±0.33 |
| Benzoic acid | F46 | 2.79±0.87 | 10.32±0.98 | 13.27±0.85 | 4.18±0.12 | 6.21±0.13 | 5.47±0.87 | ND | | 15.55±0.68 |
| 4-methylvaleric acid | F47 | 4.06±0.77 | 2.43±0.53 | 3.50±0.14 | 0.70±0.04 | 0.87±0.07 | 1.09±0.18 | 1.23±0.34 | | 10.81±0.02 |
| acetic acid | F48 | 176.65±22.20 | 263.83±49.52 | 336.79±16.29 | 112.24±16.64 | 158.22±17.14 | 195.99±7.59 | 83.19±4.31 | | 161.84±3.63 |
| 2-Hydroxy-2-methylpropyl propionate | F49 | 1.19±0.14 | 10.30±0.28 | 13.59±0.76 | 3.87±0.55 | 5.23±0.89 | 6.34±0.97 | 1.39±0.46 | | 9.74±0.26 |
| 3-Methylbutanoic acid | F50 | 91.96±22.90 | 83.89±13.13 | 97.67±4.66 | 28.53±5.07 | 34.65±5.57 | 37.79±9.63 | 35.30±1.18 | | 52.07±6.07 |
| Hydrocinnamic Acid | F51 | 0.14±0.03 | ND | ND | 0.11±0.01 | 0.21±0.02 | 0.11±0.01 | ND | | 2.69±0.02 |
| hexanoic acid | F52 | 181.00±35.98 | 135.34±22.47 | 168.42±3.67 | 50.08±2.99 | 61.11±10.20 | 57.36±4.33 | 60.62±2.58 | | ND |
| bitter | F53 | 30.07±4.95 | 23.72±4.85 | 28.87±0.82 | 10.72±2.69 | 12.19±0.70 | 9.29±0.46 | 7.10±0.43 | | 77.74±1.39 |
| Nonanoic acid | F54 | 3.36±0.74 | 5.69±1.24 | 8.00±0.91 | 5.22±1.28 | 5.81±0.46 | 2.84±0.54 | ND | | 5.66±0.24 |
| decanoic acid | F55 | ND | 2.36±0.57 | 2.12±0.45 | 2.01±0.66 | 3.14±0.50 | 1.43±0.24 | ND | | 6.35±0.61 |
| Heptenoic acid | F56 | 23.47±4.06 | 10.89±2.81 | 12.14±0.50 | 4.10±0.31 | 4.87±0.37 | 3.61±0.23 | 4.06±0.67 | | 85.60±4.28 |
| Valproic acid | F57 | 27.99±6.00 | 13.32±2.56 | 15.18±0.51 | 4.35±0.92 | 5.05±0.78 | 4.59±0.29 | 6.85±0.25 | | 141.09±25.73 |
| butyrate | F58 | 35.58±8.05 | 28.55±5.64 | 33.39±0.93 | 9.02±1.69 | 10.00±1.53 | 10.32±2.03 | 11.82±0.12 | | 163.91±23.90 |
| Phenylacetic acid | F59 | 2.25±0.16 | 7.06±0.82 | 8.34±0.12 | 2.66±0.75 | 4.44±0.36 | 3.45±0.83 | 0.57±0.05 | | 12.86±0.95 |
| homovanillic acid | F60 | ND | 0.17±0.02 | 0.15±0.01 | 0.07±0.02 | 0.13±0.01 | 0.11±0.01 | ND | | ND |
| 3-Furoxaldehyde | F61 | 2.05±0.82 | 3.31±0.51 | 3.99±0.16 | 0.67±0.13 | 0.96±0.18 | 1.02±0.16 | 2.17±0.18 | | 3.46±0.39 |
| nonanal | F62 | 2.56±0.42 | ND | ND | 4.79±0.65 | 3.22±0.06 | 3.01±0.70 | 3.05±0.80 | | ND |
| phenylacetaldehyde | F63 | 6.33±1.41 | 10.71±1.23 | 14.80±2.77 | 2.46±0.51 | 4.74±0.90 | 2.77±0.46 | 3.20±0.77 | | 12.99±2.69 |
| vanillin | F64 | 0.30±0.02 | 0.76±0.08 | 0.69±0.05 | 0.32±0.09 | 0.57±0.12 | 0.48±0.03 | ND | | 0.71±0.08 |
| N-methyl-2-pyrroleformaldehyde | F65 | 0.33±0.07 | 0.46±0.03 | 0.52±0.04 | 0.21±0.03 | 0.27±0.05 | 0.28±0.03 | 0.45±0.19 | | ND |
| benzaldehyde | F66 | 15.04±3.35 | 17.09±0.48 | 25.29±0.42 | 9.97±0.22 | 12.29±1.87 | 12.17±0.85 | 10.93±2.32 | | 35.82±5.37 |
| 3-hydroxy-4-methoxybenzaldehyde | F67 | 6.59±0.31 | ND | 8.41±0.34 | 4.52±0.49 | ND | 5.06±0.51 | 3.17±0.13 | | ND |
| 3-methylthiopropanol | F68 | 1.92±0.49 | 4.23±0.49 | 5.32±0.25 | 1.93±0.22 | 2.41±0.70 | ND | 2.59±0.01 | | 6.32±0.55 |
| β - Ethylphenylethanol | F69 | 3.24±0.58 | 3.01±0.53 | 4.13±0.21 | 1.37±0.28 | 1.47±0.25 | 1.41±0.14 | 3.74±0.27 | | 3.31±0.58 |
| 4-phenyl-3-buten-2-ol | F70 | 3.69±0.53 | 6.07±0.93 | 5.05±0.37 | 2.44±0.62 | 2.55±0.41 | 1.83±0.60 | 4.45±0.42 | | ND |
| 1-Nonanol | F71 | 0.60±0.16 | 1.87±0.00 | 6.25±0.95 | 2.21±0.67 | 2.28±0.40 | 2.41±0.11 | 1.57±0.52 | | 3.43±0.31 |
| 3-methylacetic acid 1-butanol | F72 | 3.68±0.13 | 5.56±0.64 | 9.22±0.32 | 10.51±1.42 | 11.86±1.31 | 12.93±3.33 | 4.61±0.63 | | 6.55±0.59 |
| 3-Phenylpropanol | F73 | 0.43±0.06 | 0.81±0.10 | 1.18±0.03 | 0.38±0.10 | 0.47±0.10 | 0.46±0.06 | 0.45±0.09 | | 1.86±0.23 |
| β - Methylphenylethanol | F74 | 0.36±0.09 | 0.47±0.07 | 0.67±0.00 | 0.21±0.06 | 0.24±0.04 | 0.15±0.01 | 0.39±0.11 | | 0.41±0.36 |
| 1-Octanol | F75 | 3.71±0.17 | 5.14±0.57 | 7.51±1.57 | 3.45±0.49 | 4.52±0.85 | 3.56±0.97 | 2.56±0.88 | | 10.55±1.46 |
| 1-hexanol | F76 | 8.24±0.29 | 18.05±0.80 | 22.88±4.34 | 8.36±1.30 | 11.74±1.57 | ND | 10.07±2.57 | | 56.96±5.34 |
| 2-ethylhexanol | F77 | 0.59±0.29 | 1.29±0.00 | 1.41±0.30 | 0.56±0.05 | 0.67±0.24 | 0.43±0.38 | 0.57±0.16 | | ND |
| BENZYL ALCOHOL | F78 | 9.24±1.57 | 12.72±1.79 | 16.37±0.32 | 6.04±1.37 | 7.10±1.20 | 8.34±0.72 | 9.38±2.93 | | 13.75±1.55 |
| 2-Furamol | F79 | ND | 30.34±4.18 | 36.18±0.85 | 11.47±2.23 | 14.53±2.16 | 9.58±0.41 | 26.00±0.75 | | 26.58±3.24 |
| 3-none-1-ol | F80 | ND | 1.78±0.36 | 2.74±0.27 | ND | 1.28±0.21 | 0.63±0.55 | ND | | ND |
| Furamol 2Me derivatives | F81 | ND | ND | 3.04±0.19 | 0.51±0.04 | 1.45±0.24 | 1.74±0.24 | ND | | 2.95±0.56 |
| 2,4-di-tert-butylphenol | F82 | 0.76±0.67 | 1.91±0.80 | 3.55±1.37 | 1.24±0.47 | 1.19±0.95 | ND | 0.76±0.08 | | 0.71±0.06 |
| 2-methoxy-4-vinylphenol | F83 | 8.75±1.17 | 6.53±1.19 | 8.86±0.54 | 2.44±0.61 | 2.95±0.51 | 3.16±0.10 | 7.03±0.10 | | 10.90±1.73 |
| 4-Ethyl-2-methoxyphenol | F84 | 7.32±1.23 | 14.69±2.50 | 25.75±0.46 | 7.821.65± | 7.26±1.50 | 4.88±1.82 | 4.90±1.66 | | 30.59±4.40 |
| 4-Ethylphenol | F85 | 5.20±0.87 | 35.48±6.02 | 39.36±0.90 | 8.34±1.92 | 13.92±2.64 | 6.59±0.60 | 2.49±0.83 | | 13.37±1.84 |
| 2-methoxy-5-methylphenol | F86 | 11.93±2.04 | 36.37±5.42 | 74.54±1.60 | 26.07±1.88 | 15.68±3.45 | 10.57±0.72 | 3.19±0.95 | | 19.09±3.66 |
| phenol | F87 | 3.15±0.60 | 6.23±0.86 | 8.24±0.09 | 2.40±0.55 | 3.17±0.49 | 3.07±0.28 | 2.29±0.74 | | 11.17±2.06 |
| p-cresol | F88 | 11.95±1.80 | 5.46±0.92 | 7.83±0.12 | 2.68±0.68 | 1.68±0.42 | 2.58±0.27 | 1.67±0.15 | | 63.38±9.80 |
| 2-methoxyphenol | F89 | 4.02±0.66 | 42.53±6.26 | 59.95±1.19 | 12.95±2.69 | 21.45±3.36 | 17.65±1.31 | 4.33±1.39 | | 7.67±1.36 |
| Eugenol | F90 | ND | 0.67±0.14 | 1.71±0.11 | 0.26±0.05 | 0.52±0.13 | 0.42±0.09 | ND | | ND |
| Pentadecane | F91 | 1.58±0.59 | 2.61±0.39 | 4.72±0.05 | 0.49±0.53 | 1.16±0.33 | ND | 1.09±0.32 | | 2.26±0.56 |
| Octamethylcyclopentasiloxane | F92 | 1.06±0.82 | 1.47±0.21 | 1.60±0.18 | 0.49±0.18 | 0.66±0.27 | 0.34±0.03 | 0.36±0.03 | | 2.39±0.07 |
| Hexamethylcyclooctasiloxane | F93 | 8.26±0.51 | 16.16±1.17 | 21.41±2.53 | 3.18±1.07 | 3.94±0.94 | 1.39±0.76 | 3.21±0.10 | | 13.16±0.09 |
| octamethylcyclotetrasiloxane | F94 | 17.05±2.52 | ND | ND | 13.14±5.13 | ND | 10.44±0.95 | 9.64±0.65 | | 49.89±5.05 |
| hexadecane | F95 | 0.96±0.87 | 1.50±0.63 | 4.29±0.14 | 0.76±0.21 | 1.10±0.10 | 0.57±0.02 | 0.88±0.07 | | 1.78±0.55 |
| decamethylcyclopentasiloxane | F96 | 6.73±0.79 | 3.78±0.48 | 17.60±3.55 | 4.85±0.18 | 7.44±0.91 | 6.56±0.12 | 6.52±0.65 | | 12.91±3.95 |
| Dodecarboxyl cyclohexyl siloxane | F97 | 50.78±13.45 | 84.54±8.70 | 47.00±9.63 | 14.31±5.80 | 21.59±1.57 | 7.68±0.53 | 23.33±2.41 | | 60.84±15.61 |
| Tetradecyl cycloheptylsiloxane | F98 | 47.24±2.08 | 155.26±5.23 | 158.44±5.02 | 14.02±1.12 | 24.50±1.66 | 7.21±0.45 | 26.50±3.63 | | 55.21±9.98 |
| 1,2-Dimethyl cis cyclopentane | F99 | ND | 4.21±0.30 | 3.53±0.14 | 2.49±0.36 | 2.00±0.74 | 1.87±0.62 | 1.90±0.75 | | 5.40±0.73 |
| Trimethylpyrazine | F100 | 1.75±0.11 | 2.83±0.35 | 4.22±0.19 | 0.95±0.11 | 0.87±0.69 | 0.83±0.72 | 1.02±0.25 | | ND |
| Tetramethylpyrazine | F101 | 1.42±0.31 | 1.70±0.38 | 1.47±0.23 | 0.71±0.24 | 0.75±0.21 | ND | 1.16±0.43 | | 1.35±0.40 |

Tab S-7 Eighty-eight flavor compounds identified by principal component analysis in the experimental group

| Number | Compound | Number | Compound | Number | Compound | Number | Compound | Number | Compound |
| --- | --- | --- | --- | --- | --- | --- | --- | --- | --- |
| C1 | butyrolactone | C19 | Diethyl pimelate | C37 | 2-Hydroxy-4-methylpentanoic acid ethyl ester | C55 | Phenylacetic acid | C73 | 4-vinyl-2-methoxyphenol |
| C2 | dibutyl phthalate | C20 | Ethyl 3-phenylpropionate | C38 | Phenylethyl Acetate | C56 | 3-Furoxaldehyde | C74 | 4-Ethyl-2-methoxyphenol |
| C3 | Diethyl glutarate | C21 | butyl salicylate | C39 | Ethyl phenylacetate | C57 | phenylacetaldehyde | C75 | 4-Ethylphenol |
| C4 | ethyl benzoate | C22 | 3-methylthiopropionic acid ethyl ester | C40 | Ethyl thioacetate | C58 | N-methyl-2-pyrroleformaldehyde | C76 | 2-methoxy-5-methylphenol |
| C5 | isobutyl salicylate | C23 | Dimethyl phthalate | C41 | Hept-4-ylisobutyl phthalate | C59 | benzaldehyde | C77 | phenol |
| C6 | Caprolactone | C24 | butyl lactate | C42 | 2-methylpropionic acid | C60 | 3-hydroxy-4-methoxybenzaldehyde | C78 | p-cresol |
| C7 | Ethyl hexadecanoate | C25 | Ethyl tetradecanoate | C43 | 4-methylvaleric acid | C61 | 3-methylthiopropanol | C79 | 2-methoxyphenol |
| C8 | Diethyl azelaite | C26 | butyl acetate | C44 | acetic acid | C62 | β - Ethylphenylethanol | C80 | Pentadecane |
| C9 | furfuryl acetate | C27 | ethyl caproate | C45 | 3-Methylbutanoic acid | C63 | 1-Nonanol | C81 | Octamethylcyclopentasiloxane |
| C10 | 1,2-propanediol, 1-acetate | C28 | diethyl succinate | C46 | hexanoic acid | C64 | 3-Phenylpropanol | C82 | Hexamethylcyclooctasiloxane |
| C11 | propyl lactate | C29 | Octyl salicylate | C47 | heptanoic acid | C65 | 2-phenyl-1-propanol | C83 | hexamethylcyclotrisiloxane |
| C12 | 9-Hexadecenoic acid ethyl ester | C30 | ethyl oleate | C48 | Valproic acid | C66 | Hexanol | C84 | decamethylcyclopentasiloxane |
| C13 | ethyl linoleate | C31 | Ethyl Stearate | C49 | butyrate | C67 | 2-ethylhexanol | C85 | Dodecarboxyl cyclohexyl siloxane |
| C14 | Ethyl 2-furancarboxylate | C32 | ethyl decanoate | C50 | Benzoic acid | C68 | BENZYL ALCOHOL | C86 | Tetradecyl cycloheptylsiloxane |
| C15 | ethyl valerate | C33 | Ethyl octanoate | C51 | 2-Hydroxy-2-methylpropyl propionate | C69 | 4-phenyl-3-buten-2-ol | C87 | Trimethylpyrazine |
| C16 | Ethyl pentadecanoate | C34 | ethyl enanthate | C52 | decanoic acid | C70 | 3-methylacetic acid 1-butanol | C88 | Tetramethylpyrazine |
| C17 | N-acetyl-L-leucine ethyl ester | C35 | Ethyl Butyrate | C53 | bitter | C71 | 1-Octanol |  | 4-vinyl-2-methoxyphenol |
| C18 | Diethyl phthalate | C36 | C-nonanolactone | C54 | Nonanoic acid | C72 | 2,4-di-tert-butylphenol |  |  |

Tab S-8 Eighty-three flavor compounds identified by principal component analysis in the control group

| Number | Compound | Number | Compound | Number | Compound | Number | Compound | Number | Compound |
| --- | --- | --- | --- | --- | --- | --- | --- | --- | --- |
| D1 | ethyl benzoate | D18 | Dimethyl Phthalate | D35 | 9-Tetradecenoic acid ethyl ester | D52 | N-methyl-2-pyrroleformaldehyde | D69 | 4-Ethylphenol |
| D2 | dibutyl phthalate | D19 | Ethyl Myristate | D36 | 2-methylpropionic acid | D53 | benzaldehyde | D70 | 2-methoxy-5-methylphenol |
| D3 | isobutyl salicylate | D20 | butyl acetate | D37 | Benzoic acid | D54 | 3-methylthiopropanol | D71 | phenol |
| D4 | Diethyl glutarate | D21 | ethyl caproate | D38 | 4-methylvaleric acid | D55 | β - Ethylphenylethanol | D72 | p-cresol |
| D5 | Isocyanates | D22 | diethyl succinate | D39 | acetic acid | D56 | 4-phenyl-3-buten-2-ol | D73 | 2-methoxyphenol |
| D6 | Ethyl hexadecanoate | D23 | ethyl oleate | D40 | 2-Hydroxy-2-methylpropyl propionate | D57 | 1-Nonanol | D74 | Pentadecane |
| D7 | Diethyl azelaite | D24 | Ethyl octadecanoate | D41 | 3-Methylbutanoic acid | D58 | 3-methylacetic acid 1-butanol | D75 | Octamethylcyclopentasiloxane |
| D8 | 2-furanmethanol acetate | D25 | ethyl decanoate | D42 | hexanoic acid | D59 | 3-Phenylpropanol | D76 | Hexamethylcyclooctasiloxane |
| D9 | 9-Hexadecenoic acid ethyl ester | D26 | Ethyl octanoate | D43 | bitter | D60 | β - Methylphenylethanol | D77 | hexadecane |
| D10 | ethyl linoleate | D27 | ethyl enanthate | D44 | Nonanoic acid | D61 | 1-Octanol | D78 | decamethylcyclopentasiloxane |
| D11 | Ethyl 4-oxovalerate | D28 | Ethyl Butyrate | D45 | Heptenoic acid | D62 | 1-hexanol | D79 | Dodecarboxyl cyclohexyl siloxane |
| D12 | Ethyl pentadecanoate | D29 | C-nonanolactone | D46 | Valproic acid | D63 | 2-ethylhexanol | D80 | Tetradecyl cycloheptylsiloxane |
| D13 | Diethyl phthalate | D30 | 2-Hydroxy-4-methylpentanoic acid ethyl ester | D47 | butyrate | D64 | BENZYL ALCOHOL | D81 | 1,2-Dimethyl cis cyclopentane |
| D14 | Diethyl pimelate | D31 | Phenylethyl Acetate | D48 | Phenylacetic acid | D65 | 2-Furamol | D82 | Trimethylpyrazine |
| D15 | Ethyl phenylpropionate | D32 | Ethyl phenylacetate | D49 | 3-Furoxaldehyde | D66 | 2,4-di-tert-butylphenol | D83 | Tetramethylpyrazine |
| D16 | 2-Hydroxy-3-phenylpropanoic acid ethyl ester | D33 | 3- (Methylthio) acetate propyl ester | D50 | phenylacetaldehyde | D67 | 2-methoxy-4-vinylphenol |  | 4-Ethylphenol |
| D17 | butyl lactate | D34 | Hept-4-ylisobutyl phthalate | D51 | vanillin | D68 | 4-Ethyl-2-methoxyphenol |  |  |

Tab S-9 Summary of bacterial sequencing data diversity indices

|  | Sample | Chao1 | Simpson | Shannon | Pieloue | Observed speciess | Goods coverage |
| --- | --- | --- | --- | --- | --- | --- | --- |
| Experimental group | A1 | 4179.12 | 0.998013 | 10.5419 | 0.877106 | 4150.3 | 0.993852 |
|  | A4 | 624.004 | 0.873028 | 4.40556 | 0.479024 | 586.9 | 0.99758 |
|  | A7 | 3262.93 | 0.911118 | 7.26652 | 0.626678 | 3094.1 | 0.987318 |
|  | A12 | 1778.14 | 0.876862 | 5.58209 | 0.523922 | 1611.8 | 0.992074 |
|  | A17 | 1530.93 | 0.820683 | 4.8077 | 0.459734 | 1406.2 | 0.993478 |
|  | A27 | 1092.81 | 0.714854 | 3.60635 | 0.360519 | 1026.3 | 0.996208 |
|  | A42 | 1178.37 | 0.894808 | 5.30513 | 0.524608 | 1107.1 | 0.995258 |
|  | A62 | 93.1346 | 0.587788 | 1.83549 | 0.288036 | 82.9 | 0.999611 |
| Control group | C1 | 1619.32 | 0.700619 | 3.756 | 0.354743 | 1539.2 | 0.995205 |
|  | C4 | 283.118 | 0.785833 | 3.32463 | 0.410656 | 273.6 | 0.999298 |
|  | C7 | 2247.55 | 0.85204 | 5.93438 | 0.534165 | 2209.7 | 0.996188 |
|  | C12 | 1663.99 | 0.831432 | 5.35879 | 0.501701 | 1642 | 0.997487 |
|  | C17 | 636.794 | 0.663691 | 2.9084 | 0.313852 | 616 | 0.998533 |
|  | C27 | 2855.24 | 0.919742 | 7.50144 | 0.654052 | 2835.1 | 0.99688 |
|  | C42 | 1907.03 | 0.88827 | 5.92566 | 0.544913 | 1877.4 | 0.996799 |
|  | C62 | 1039.16 | 0.737368 | 4.05894 | 0.406889 | 1006.8 | 0.997772 |

Tab S-10 Summary of fungal sequencing data diversity indices

|  | Sample | Chao1 | Simpson | Shannon | Pieloue | Observed speciess | Goods coverage |
| --- | --- | --- | --- | --- | --- | --- | --- |
| Experimental group | A1 | 219.423 | 0.830467 | 3.79695 | 0.488367 | 219 | 0.999942 |
|  | A4 | 177.205 | 0.836235 | 3.68956 | 0.4944 | 176.4 | 0.99993 |
|  | A7 | 237.997 | 0.875093 | 4.09746 | 0.519086 | 237.8 | 0.999968 |
|  | A12 | 147.156 | 0.949505 | 5.07271 | 0.704672 | 146.9 | 0.999964 |
|  | A17 | 142 | 0.947249 | 5.13531 | 0.71825 | 142 | 0.999996 |
|  | A27 | 84.9827 | 0.841026 | 3.26871 | 0.510123 | 84.9 | 0.999976 |
|  | A42 | 116.925 | 0.774206 | 3.38198 | 0.492613 | 116.6 | 0.999969 |
|  | A62 | 72.9013 | 0.768733 | 2.7029 | 0.437798 | 72.2 | 0.999954 |
| Control group | C1 | 152.206 | 0.908394 | 4.45713 | 0.614953 | 152 | 0.999971 |
|  | C4 | 115.037 | 0.91016 | 4.2206 | 0.616552 | 115 | 0.999991 |
|  | C7 | 160.181 | 0.94026 | 4.9221 | 0.67224 | 160 | 0.999978 |
|  | C12 | 164.218 | 0.944841 | 5.08319 | 0.69088 | 164 | 0.999975 |
|  | C17 | 122.076 | 0.949258 | 4.86111 | 0.701504 | 121.9 | 0.999979 |
|  | C27 | 180.468 | 0.880325 | 4.22102 | 0.563717 | 179.5 | 0.999958 |
|  | C42 | 188.026 | 0.901626 | 4.30348 | 0.569826 | 187.7 | 0.999974 |
|  | C62 | 202.342 | 0.922787 | 4.64815 | 0.607348 | 201.3 | 0.999924 |

Tab S-11 Correlation of Fflavor compounds with physicochemical indicators, enzyme activity, and microbial community in the experimental group

| **Compounds** |  | **Correlation with** | **p-value** | **r** |
| --- | --- | --- | --- | --- |
| butyrolactone | C1 | Thermoascus、Aspergillus、Lodderomyces | 0.013、0.006、0 | 0.920、0.859、0.960 |
| dibutyl phthalate | C2 | Cellulase, α- amylase、Rhodotorula、Cutaneotrichosporon | 0.045、0.046、0.046、0.042 | 0.717、0.716、-0.716、-0.726 |
| Diethyl glutarate | C3 | Thermoascus、Aspergillus、Lodderomyces | 0.003、0.004、0 | 0.888、0.880、0.967 |
| ethyl benzoate | C4 | Moisture、starch、reducing sugar、acidity、α- amylase、acid protease、Lactobacillus、Thermoascus、Aspergillus、Lodderomyces | 0.027、0.001、0.019、0.002、0.049、0.001、0.0320.021、0.001、0.015 | 0.764、-0.919、-0.792、0.901、-0.709、0.938、0.749、0.783、0.938、0.808 |
| isobutyl salicylate | C5 | Moisture、starch、reducing sugar、acidity、acid protease、Lactobacillus、Thermoascus、Aspergillus、Lodderomyces | 0.025、0.001、0.012、0.002、0.006、0.032、0.013、0、0.003 | 0.770、-0.914、-0.824、0.897、0.858、0.750、0.821、0.957、0.892 |
| Caprolactone | C6 | A4b | 0.021 | -0.784 |
| Ethyl hexadecanoate | C7 | Moisture、starch、reducing sugar、acidity、acid protease、Lactobacillus、Methylobacter、Subgroup_10、SBR1031、A4b、Bacteroidetes_vadinHA17、Thermoascus、Aspergillus | 0.011、0.027、0.032、0.032、0.038、0.003、0.043、0.019、0.026、0.023、0.027、0.014、0.024 | 0.830、-0.764、-0.750、0.779、0.734、0.889、-0.722、-0.792、-0.769、-0.777、-0.764、0.812、0.773 |
| Diethyl azelaite | C8 | Thermoascus、Aspergillus、Lodderomyces | 0.005、0.013、0.000 | 0.871、0.820、0.947 |
| furfuryl acetate | C9 | Thermoascus、Aspergillus、Lodderomyces | 0.000、0.049、0.044 | 0.941、0.708、0.719 |
| 1,2-propanediol, 1-acetate | C10 | Moisture、starch、reducing sugar、acidity、acid protease、Lactobacillus、Thermoascus、Aspergillus、Lodderomyces | 0.008、0、0.003、0.001、0.011、0.043、0.047、0.002、0.009 | 0.849、-0.948、-0.895、0.935、0.831、0.721、0.713、0.901、0.940 |
| propyl lactate | C11 | starch、acidity、Thermoascus、Aspergillus、Lodderomyces | 0.03、0.036、0.007、0.002、0.000 | -0.756、0.739、0.851、0.910、0.977 |
| 9-Hexadecenoic acid ethyl ester | C12 | Moisture、Lactobacillus、Thermoascus、Aspergillus、Lodderomyces | 0.034、0.024、0.014、0.004、0.031 | 0.744、0.774、0.812、0.729、0.753 |
| ethyl linoleate | C13 | Lactobacillus、Thermoascus、Aspergillus、Lodderomyces1 | 0.018、0.000、0.004、0.012 | 0.795、0.943、0.874、0.822 |
| Ethyl 2-furancarboxylate | C14 | starch、reducing sugar、acidity、Lactobacillus、Thermoascus、Aspergillus、Lodderomyces | 0.011、0.033、0.013、0.043、0.008、0.001、0.000 | -0.831、-0.748、0.816、0.723、0.847、0.936、0.954 |
| Ethyl pentadecanoate | C16 | Moisture、starch、reducing sugar、acidity、Lactobacillus、Subgroup_10、Thermoascus、Aspergillus、Lodderomyces | 0.042、0.03、0.043、0.028、0.006、0.031、0.002、0.005、0.014 | 0.724、-0.757、-0.721、0.762、0.862、-0.752、0.913、0.871、0.815 |
| N-acetyl-L-leucine ethyl ester | C17 | Thermoascus、Aspergillus、Lodderomyces | 0.006、0.001、0.000 | 0.864、0.917、0.947 |
| Diethyl phthalate | C18 | Thermoascus、Aspergillus、Lodderomyces | 0.005、0.022、0.001 | 0.866、0.783、0.925 |
| Diethyl pimelate | C19 | Thermoascus、Aspergillus、Lodderomyces | 0.005、0.017、0.001 | 0.868、0.800、0.936 |
| Ethyl 3-phenylpropionate | C20 | Thermoascus、Aspergillus、Lodderomyces | 0.002、0.02、0.003 | 0.907、0.789、0.892 |
| butyl salicylate | C21 | starch、acidity、Lactobacillus、Thermoascus、Aspergillus、Lodderomyces | 0.029、0.034、0.043、0.002、0.001、0.000 | -0.759、0.746、0.723、0.909、0.904、0.959 |
| 3-methylthiopropionic acid ethyl ester | C22 | Thermoascus、Aspergillus、Lodderomyces | 0.006、0.002、0.000 | 0.863、0.898、0.944 |
| Dimethyl phthalate | C23 | starch、acidity、Thermoascus、Aspergillus、Lodderomyces | 0.04、0.046、0.007、0.001、0.000 | -0.730、0.715、0.853、0.919、0.965 |
| butyl lactate | C24 | Thermoascus、Aspergillus、Lodderomyces | 0.001、0.001、0.000 | 0.935、0.923、0.951 |
| Ethyl tetradecanoate | C25 | Moisture、starch、reducing sugar、acidity、Lactobacillus、Subgroup_10、Bacteroidetes_vadinHA17、Thermoascus、Aspergillus、Lodderomyces | 0.028、0.025、0.035、0.023、0.007、0.026、0.007、0.005、0.008、0.017 | 0.762、-0.771、-0.743、-0.779、0.854、-0.769、-0.722、0.869、0.846、0.799 |
| ethyl caproate | C27 | Thermoascus | 0.041 | 0.726 |
| diethyl succinate | C28 | Thermoascus、Aspergillus、Lodderomyces | 0.005、0.004、0.000 | 0.870、0.882、0.959 |
| Octyl salicylate | C29 | Starmerella | 0.021 | 0.784 |
| ethyl oleate | C30 | Lactobacillus、Thermoascus、Aspergillus、Lodderomyces | 0.039、0.007、0.028、0.012 | 0.732、0.856、0.763、0.825 |
| Ethyl Stearate | C31 | Lactobacillus、Thermoascus、Aspergillus、Lodderomyces | 0.014、0.000、0.008、0.015 | 0.814、0.954、0.844、0.809 |
| ethyl decanoate | C32 | Lactobacillus、Methylobacter、Subgroup_10、SBR1031、Kroppenstedtia、Macellibacteroides、A4b、Bacteroidetes_vadinHA17 | 0.046、0.035、0.042、0.016、0.036、0.009、0.036 | 0.715、-0.743、-0.724、-0.807、-0.740、-0.843、-0.738 |
| Ethyl octanoate | C33 | Thermoascus、Aspergillus、Lodderomyces | 0.000、0.016、0.02 | 0.970、0.805、0.790 |
| ethyl enanthate | C34 | Thermoascus、Lodderomyces | 0.024、0.035 | 0.775、0.743 |
| Ethyl Butyrate | C35 | Thermoascus、Aspergillus、Lodderomyces | 0.007、0.006、0.000 | 0.854、0.864、0.971 |
| C-nonanolactone | C36 | starch、acidity、acid protease、Lactobacillus、Thermoascus、Aspergillus、Lodderomyces | 0.027、0.032、0.033、0.033、0.001、0.000、0.001 | -0.765、0.752、0.748、0.748、0.927、0.944、0.925 |
| 2-Hydroxy-4-methylpentanoic acid ethyl ester | C37 | starch、reducing sugar、acidity、acid protease、Lactobacillus、Thermoascus、Aspergillus、Lodderomyces | 0.011、0.035、0.014、0.034、0.035、0.004、0.000、0.000 | -0.827、-0.741、0.812、0.744、0.741、0.882、0.955、0.954 |
| Ethyl thioacetate | C40 | Thermoascus、Aspergillus、Lodderomyces | 0.012、0.009、0.000 | 0.824、0.843、0.966 |
| Hept-4-ylisobutyl phthalate | C41 | cellulase、Acinetobacter、Thauera、Methylobacter、SBR1031、Macellibacteroides、A4b、Bacteroidetes_vadinHA17、Pseudallescheria | 0.045、0.026、0.03、0.019、0.019、0.021、0.037、0.036、0.033 | 0.717、0.768、0.755、0.794、0.791、0.785、0.738、0.740、0.747 |
| 2-methylpropionic acid | C42 | Kroppenstedtia、Starmerella | 0.016、0.012 | -0.807、-0.825 |
| 4-methylvaleric acid | C43 | Thermoascus、Lodderomyces | 0.011、0.013 | 0.830、0.819 |
| acetic acid | C44 | Moisture、starch、reducing sugar、acidity、acid protease、Aspergillus | 0.002、0.011、0.017、0.012、0.005、0.044 | 0.904、-0.830、-0.800、0.826、0.868、0.720 |
| 3-Methylbutanoic acid | C45 | Thermoascus、Aspergillus、Lodderomyces | 0.01、0.017、0.018 | 0.837、0.799、0.797 |
| hexanoic acid | C46 | Acetobacter、Xeromyces | 0.036、0.041 | 0.739、0.726 |
| heptanoic acid | C47 | Thermoascus、Aspergillus、Lodderomyces | 0.019、0.018、0.001 | 0.791、0.798、0.937 |
| butyrate | C49 | Thermoascus、Lodderomyces | 0.019、0.029 | 0.792、0.758 |
| Benzoic acid | C50 | starch、reducing sugar、acidity、acid protease、Lactobacillus、Thermoascus、Aspergillus、Lodderomyces | 0.011、0.035、0.014、0.016、0.03、0.002、0.000、0.001 | -0.830、-0.743、0.812、0.806、0.756、0.912、0.963、0.914 |
| 2-Hydroxy-2-methylpropyl propionate | C51 | starch、reducing sugar、acidity、acid protease、Lactobacillus、Thermoascus、Aspergillus、Lodderomyces | 0.011、0.046、0.014、0.034、0.034、0.006、0.000、0.000 | -0.827、-0.715、0.815、0.745、0.745、0.865、0.952、0.961 |
| decanoic acid | C52 | starch、acidity、acid protease、Lactobacillus、Thermoascus、Aspergillus、Lodderomyces | 0.024、0.031、0.01、0.05、0.004、0.001、0.005 | -0.773、0.752、0.835、0.707、0.883、0.930、0.870 |
| bitter | C53 | starch、reducing sugar、acidity、acid protease、Thermoascus、Aspergillus、Lodderomyces | 0.007、0.035、0.009、0.032、0.043、0.001、0.000 | -0.857、-0.742、0.839、0.749、0.722、0.918、0.945 |
| Nonanoic acid | C54 | starch、acidity、acid protease、Lactobacillus、Thermoascus、Aspergillus、Lodderomyces | 0.031、0.038、0.009、0.043、0.009、0.008、0.024 | -0.752、0.736、0.841、0.722、0.838、0.846、0.776 |
| Phenylacetic acid | C55 | Moisture、starch、reducing sugar、acidity、acid protease、Lactobacillus、Thermoascus、Aspergillus、Lodderomyces | 0.04、0.006、0.02、0.008、0.008、0.03、0.003、0.000、0.003 | 0.729、-0.863、-0.788、0.844、0.847、0.757、0.891、0.972、0.894 |
| 3-Furoxaldehyde | C56 | reducing sugar、Thermoascus、Aspergillus、Lodderomyces | 0.044、0.043、0.017、0.012 | -0.721、-0.722、0.801、0.826 |
| phenylacetaldehyde | C57 | starch、acidity、acid protease、Thermoascus、Aspergillus、Lodderomyces | 0.027、0.037、0.043、0.003、0.001、0.001 | -0.765、0.737、0.721、0.887、0.936、0.934 |
| N-methyl-2-pyrroleformaldehyde | C58 | Subgroup_10 | 0.03 | -0.757 |
| benzaldehyde | C59 | Thermoascus、Aspergillus、Lodderomyces | 0.000、0.001、0.000 | 0.946、0.916、0.943 |
| 3-methylthiopropanol | C61 | Moisture、starch、reducing sugar、acidity、acid protease、Thermoascus、Aspergillus | 0.018、0.027、0.03、0.031、0.013、0.037、0.012 | 00.796、-0.765、-0.757、0.754、0.820、0.738、0.824 |
| β - Ethylphenylethanol | C62 | Thermoascus、Aspergillus、Lodderomyces | 0.002、0.005、0.001 | 0.907、0.872、0.928 |
| 1-Nonanol | C63 | Thermoascus、Aspergillus、Lodderomyces | 0.016、0.024、0.001 | 0.803、0.775、0.933 |
| 3-Phenylpropanol | C64 | BIrii41、Thermoascus | 0.05、0.001 | -0.707、0.922 |
| 2-ethylhexanol | C67 | Aspergillus、Lodderomyces | 0.028、0.028 | -0.763、-0.763 |
| BENZYL ALCOHOL | C68 | Moisture、starch、reducing sugar、acidity、acid protease、Thermoascus、Aspergillus、Lodderomyces | 0.048、0.01、0.017、0.016、0.011、0.009、0.001、0.007 | 0.711、-0.836、-0.802、0.806、0.828、0.838、0.934、0.855 |
| 4-phenyl-3-buten-2-ol | C69 | Moisture、starch、acidity、cellulase、α- amylase、acid protease、Lactobacillus、Thauera、Methylobacter、SBR1031、Macellibacteroides、A4b、Thermoascus、Aspergillus、Pseudallescheria | 0.042、0.021、0.019、0.035、0.033、0.007、0.017、0.033、0.018、0.021、0.029、0.009、0.045、0.012、0.041 | 0.724、-0.787、0.794、-0.742、-0.748、0.857、0.799、-0.747、-0.798、-0.786、-0.760、-0.839、0.718、0.824、-0.727 |
| 3-methylacetic acid 1-butanol | C70 | Moisture、starch、acidity、acid protease | 0.009、0.041、0.038、0.005 | 0.838、-0.727、0.734、0.870 |
| 1-Octanol | C71 | starch、acidity、Lactobacillus、Thermoascus、Aspergillus、Lodderomyces | 0.028、0.026、0.018、0.011、0.004、0.001 | -0.763、0.768、0.797、0.829、0.877、0.922 |
| 2,4-di-tert-butylphenol | C72 | Bacillus、Hydrogenophaga、Kroppenstedtia、Enterobacter、Virgisporangium、Monascus、Trichosporon、Starmerella | 0.005、0.033、0.016、0.015、0.036、0.011、0.013、0.01 | -0.866、-0.749、-0.804、-0.809、-0.740、-0.829、-0.817、-0.834 |
| 4-vinyl-2-methoxyphenol | C73 | Limosilactobacillus、Kazachstania | 0.009、0.021 | -0.842、-0.783 |
| 4-Ethyl-2-methoxyphenol | C74 | starch、acidity、acid protease、Lactobacillus、Thermoascus、Aspergillus、Lodderomyces | 0.025、0.031、0.024、0.032、0.000、0.000、0.002 | -0.771、0.754、0.776、0.751、0.943、0.943、0.906 |
| 4-Ethylphenol | C75 | Moisture、starch、reducing sugar、acidity、acid protease、Lactobacillus、Thermoascus、Aspergillus、Lodderomyces | 0.045、0.019、0.045、0.023、0.01、0.027、0.001、0.001、0.01 | 0.718、-0.793、-0.717、0.777、0.833、0.765、0.922、0.917、0.834 |
| 2-methoxy-5-methylphenol | C76 | Moisture、starch、reducing sugar、acidity、acid protease、Lactobacillus、Thermoascus、Aspergillus、Lodderomyces | 0.047、0.011、0.032、0.014、0.006、0.028、0.001、0.000、0.007 | 0.713、-0.831、-0.750、0.812、0.863、0.763、0.915、0.957、0.857 |
| phenol | C77 | Thermoascus、Aspergillus、Lodderomyces | 0.002、0.007、0.004 | 0.908、0.853、0.875 |
| 2-methoxyphenol | C79 | starch、reducing sugar、acidity、acid protease、Thermoascus、Aspergillus、Lodderomyces | 0.032、0.047、0.04、0.039、0.002、0.001、0.002 | -0.750、-0.713、0.730、0.733、0.904、0.925、0.903 |
| Pentadecane | C80 | starch、acidity、cellulase、Glucosidase、α- amylase、acid protease、Lactobacillus、Acinetobacter、Thauera、Methylobacter、SBR1031、Macellibacteroides、A4b、Thermoascus、Pseudallescheria | 0.004、0.003、0.018、0.008、0.002、0.021、0.045、0.026、0.017、0.013、0.025、0.021、0.03、0.029、0.029 | 0.877、-0.886、0.796、0.848、0.910、-0.786、-0.717、0.768、0.802、0.818、0.770、0.783、0.756、-0.760、0.758 |
| Octamethylcyclopentasiloxane | C81 | Thermoascus、Aspergillus、Lodderomyces | 0.006、0.011、0.001 | 0.865、0.830、0.928 |
| Hexamethylcyclooctasiloxane | C82 | Thermoascus、Aspergillus、Lodderomyces | 0.005、0.017、0.001 | 0.872、0.800、0.921 |
| hexamethylcyclotrisiloxane | C83 | Thermoascus、Aspergillus、Lodderomyces | 0.002、0.02、0.001 | 0.906、0.835、0.935 |
| decamethylcyclopentasiloxane | C84 | Thermoascus、Aspergillus、Lodderomyces | 0.000、0.006、0.001 | 0.943、0.860、0.924 |
| Dodecarboxyl cyclohexyl siloxane | C85 | Komagataeibacter、Rummeliibacillus、Coniochaeta | 0.032、0.019 | -0.750、-0.791 |
| Trimethylpyrazine | C87 | Thermoascus、Aspergillus、Lodderomyces | 0.021、0.043、0.002、0.02 | 0.784、0.723、0.913、-0.787 |
| Tetramethylpyrazine | C88 | Moisture、 starch、acidity、acidic protease、Limosilactobacillus、Kazachstania | 0.04、0.023、0.03、0.009、0.015、0.008 | -0.729、0.777、-0.756、-0.843、0.810、0.847 |

Tab S- 12 Correlation of flavor compounds with pysicochemical indicators, enzyme activity, and microbial community in the control group

| **Compounds** |  | **Correlation with** | | **p-value** | **r** |
| --- | --- | --- | --- | --- | --- |
| isobutyl salicylate | D3 | | Knufia、Nothophoma、Neocucurbitaria、Alternaria | 0.038、0.042、0.038、0.041 | 0.735、0.724、0.735、0.727 |
| Diethyl glutarate | D4 | | Kocuria、Paracoccus、Knufia、Nothophoma、Neocucurbitaria、Alternari | 0.012、0.029、0.003、0.004、0.003、0.004 | 0.826、0.758、0.885、0.877、0.885、0.880 |
| Isocyanates | D5 | | reducing sugar、Cephalotrichum | 0.02、0.012 | 0.790、0.826 |
| Diethyl azelaite | D7 | | Knufia、Nothophoma、Neocucurbitaria、Alternari | 0.029、0.029、0.029、0.032 | 0.759、0.759、0.759、0.750 |
| Ethyl 4-oxovalerate | D11 | | acid protease、Kocuria、Paracoccus、Knufia、Nothophoma、Neocucurbitaria、Alternari | 0.041、0.02、0.045、0.008、0.005、0.008、0.009 | 0.726、0.790、0.719、0.848、0.874、0.848、0.942 |
| Diethyl pimelate | D14 | | Kocuria、Paracoccus、Knufia、Nothophoma、Neocucurbitaria、Alternari | 0.013、0.033、0.004、0.005、0.004、0.005 | 0.816、0.747、0.877、0.869、0.877、0.872 |
| Ethyl phenylpropionate | D15 | | Kocuria、Paracoccus、Knufia、Nothophoma、Neocucurbitaria、Alternari | 0.002、0.008、0.000、0.000、0.000、0.000 | 0.913、0.846、0.967、0.964、0.967、0.963 |
| 2-Hydroxy-3-phenylpropanoic acid ethyl ester | D16 | | Kocuria、Paracoccus、Knufia、Nothophoma、Neocucurbitaria、Alternari | 0.014、0.033、0.005、0.006、0.005、0.006 | 0.813、0.747、0.868、0.863、0.868、0.864 |
| butyl lactate | D17 | | Kocuria、Paracoccus、Knufia、Nothophoma、Neocucurbitaria、Alternari | 0.001、0.005、0.000、0.000、0.000、0.000 | 0.929、0.967、0.974、0.970、0.974、0.971 |
| butyl acetate | D20 | | Cephalotrichum | 0.049 | 0.709 |
| ethyl caproate | D21 | | Knufia、Nothophoma、Neocucurbitaria、Alternari | 0.04、0.044、0.04、0.048 | 0.728、0.720、0.728、0.722 |
| diethyl succinate | D22 | | Kocuria、Paracoccus、Knufia、Nothophoma、Neocucurbitaria、Alternari | 0.003、0.01、0.001、0.001、0.001、0.001 | 0.894、0.836、0.937、0.930、0.937、0.934 |
| ethyl decanoate | D25 | | Kocuria、Knufia、Nothophoma、Neocucurbitaria | 0.044、0.024、0.025、0.024 | 0.719、0.774、0.772、0.774 |
| Ethyl octanoate | D26 | | cellulase、Trichothecium、Cephalotrichum | 0.032、0.025、0.011 | -0.750、-0.772、-0.831 |
| ethyl enanthate | D27 | | Glucosidase | 0.038 | 0.734 |
| Ethyl Butyrate | D28 | | acid protease、Kocuria、Paracoccus、Knufia、Nothophoma、Neocucurbitaria、Alternari | 0.041、0.000、0.001、0.000、0.000、0.000、0.000 | 0.728、0.966、0.915、0.995、0.993、0.995、0.994 |
| Phenylethyl Acetate | D31 | | Saccharomyces | 0.05 | -0.707 |
| Hept-4-ylisobutyl phthalate | D34 | | Xeromyces | 0.047 | 0.714 |
| 9-Tetradecenoic acid ethyl ester | D35 | | Scedosporium | 0.044 | 0.720 |
| 4-methylvaleric acid | D38 | | Kocuria、Paracoccus、Knufia、Nothophoma、Neocucurbitaria、Alternari | 0.004、0.012、0.001、0.001、0.001、0.001 | 0.882、0.823、0.927、0.917、0.927、0.924 |
| acetic acid | D39 | | Corynebacterium、Scedosporium | 0.026、0.032 | -0.767、0.750 |
| 3-Methylbutanoic acid | D41 | | α-amylase | 0.032 | 0.751 |
| hexanoic acid | D42 | | Moisture、starch、reducing sugar、acidity、cellulase、α-amylase、acid protease、Lysinibacillus、Kazachstania | 0.001、0.003、0.007、0.005、0.036、0.008、0.009、0.03、0.018 | -0.934、0.895、0.857、-0.869、0.740、0.845、-0.841、0.757、0.797 |
| bitter | D43 | | Kocuria、Paracoccus、Knufia、Nothophoma、Neocucurbitaria、Alternari | 0.007、0.022、0.001、0.002、0.001、0.001 | 0.855、0.782、0.920、0.912、0.920、0.914 |
| Nonanoic acid | D44 | | Acetobacter、Bacillus、Sphingobacterium、Trichosporon、Monascus | 0.024、0.029、0.029、0.036、0.017 | -0.775、-0.760、-0.759、-0.739、-0.800 |
| Heptenoic acid | D45 | | Kocuria、Paracoccus、Knufia、Nothophoma、Neocucurbitaria、Alternari | 0.001、0.005、0.000、0.000、0.000、0.000 | 0.929、0.871、0.970、0.964、0.970、0.968 |
| Valproic acid | D46 | | Kocuria、Paracoccus、Knufia、Nothophoma、Neocucurbitaria、Alternari | 0.000、0.002、0.000、0.000、0.000、0.000 | 0.952、0.989、0.985、0.980、0.985、0.984 |
| butyrate | D47 | | Kocuria、Paracoccus、Knufia、Nothophoma、Neocucurbitaria、Alternari | 0.001、0.004、0.000、0.000、0.000、0.000 | 0.937、0.880、0.977、0.969、0.977、0.974 |
| Phenylacetic acid | D48 | | Knufia、Nothophoma、Neocucurbitaria、Alternari | 0.025、0.025、0.025、0.028 | 0.772、0.772、0.771、0.761 |
| vanillin | D51 | | Acetobacter、Bacillus、Sphingobacterium、Corynebacterium、Trichosporon、Monascus | 0.016、0.04、0.034、0.032、0.031、0.03 | -0.803、-0.730、-0.746、-0.751、-0.753、-0.757 |
| N-methyl-2-pyrroleformaldehyde | D52 | | acid protease、Knufia、Nothophoma、Neocucurbitaria、Alternari | 0.034、0.03、0.026、0.03、0.032 | -0.744、-0.757、-0.769、-0.756、-0.749 |
| benzaldehyde | D53 | | Kocuria、Paracoccus、Knufia、Nothophoma、Neocucurbitaria、Alternari | 0.023、0.049、0.01、0.011、0.01、0.01 | 0.776、0.709、0.837、0.828、0.837、0.832 |
| 4-phenyl-3-buten-2-ol | D56 | | reducing sugar、acid protease、Kazachstania | 0.022、0.037、0.017 | 0.780、-0.736、0.802 |
| 1-Nonanol | D57 | | Scedosporium、Pseudallescheria | 0.004、0.015 | 0.877、0.809 |
| 3-methylacetic acid 1-butanol | D58 | | Pichia | 0.04 | -0.730 |
| 3-Phenylpropanol | D59 | | Kocuria、Paracoccus、Knufia、Nothophoma、Neocucurbitaria、Alternari | 0.016、0.034、0.007、0.008、0.007、0.007 | 0.805、0.745、0.854、0.845、0.854、0.850 |
| β - Methylphenylethanol | D60 | | Scedosporium | 0.023 | 0.749 |
| 1-Octanol | D61 | | Kocuria、Knufia、Nothophoma、Neocucurbitaria、Alternari | 0.034、0.012、0.012、0.012、0.013 | 0.744、0.825、0.825、0.825、0.817 |
| 1-hexanol | D62 | | Kocuria、Paracoccus、Knufia、Nothophoma、Neocucurbitaria、Alternari | 0.003、0.009、0.001、0.001、0.001、0.001 | 0.891、0.841、0.921、0.919、0.921、0.920 |
| 2-ethylhexanol | D63 | | reducing sugar、cellulase、acid protease | 0.008、0.024、0.034 | 0.846、0.775、-0.745 |
| 2,4-di-tert-butylphenol | D66 | | Scedosporium、Pseudallescheria | 0.006、0.013 | 0.859、0.816 |
| 4-Ethyl-2-methoxyphenol | D68 | | Knufia、Neocucurbitaria、Alternaria | 0.046、0.046、0.049 | 0.715、0.715、0.709 |
| 4-Ethylphenol | D69 | | reducing sugar | 0.022 | 0.781 |
| 2-methoxy-5-methylphenol | D70 | | Scedosporium、Pseudallescheria | 0.003、0.005 | 0.892、0.872 |
| phenol | D71 | | Knufia、Nothophoma、Neocucurbitaria、Alternari | 0.026、0.029、0.026、0.028 | 0.767、0.758、0.767、0.761 |
| p-cresol | D72 | | Kocuria、Paracoccus、Knufia、Nothophoma、Neocucurbitaria、Alternari | 0.000、0.003、0.000、0.000、0.000、0.000 | 0.946、0.889、0.985、0.979、0.985、0.983 |
| 2-methoxyphenol | D73 | | reducing sugar、Scedosporium | 0.045、0.023 | 0.717、0.778 |
| Pentadecane | D74 | | Scedosporium | 0.014 | 0.815 |
| Octamethylcyclopentasiloxane | D75 | | Knufia、Nothophoma、Neocucurbitaria、Alternari | 0.035、0.038、0.035、0.037 | 0.743、0.734、0.743、0.736 |
| Hexamethylcyclooctasiloxane | D76 | | reducing sugar | 0.044 | 0.719 |
| hexadecane | D77 | | Scedosporium、Pseudallescheria | 0.000、0.013 | 0.944、0.819 |
| decamethylcyclopentasiloxane | D78 | | Trichothecium、Scedosporium | 0.047、0.014 | -0.712、0.813 |
| Dodecarboxyl cyclohexyl siloxane | D79 | | Cephalotrichum | 0.034 | 0.745 |
| Tetradecyl cycloheptylsiloxane | D80 | | reducing sugar、Cephalotrichum | 0.006、0.031 | 0.861、0.754 |
| Trimethylpyrazine | D82 | | reducing sugar、acid protease、Scedosporium、Pseudallescheria | 0.007、0.019、0.018、0.047 | 0.855、-0.792、0.796、0.714 |
| Tetramethylpyrazine | D83 | | Subgroup_10、A4b、MND1、OM190 | 0.029、0.03、0.039、0.021 | -0.759、-0.733、-0.785 |

Tab S-13 Correlation between physicochemical indicators and microbial community

| **Compounds** |  | **Correlation with** | | **p-value** | **r** |
| --- | --- | --- | --- | --- | --- |
| experimental group | | | | | |
| Lactobacillus | M1 | Moisture、starch、acidity、cellulase、Glucosidase、α-amylase、acid protease | | 0.021、0.022、0.015、0.016、0.009、0.009、0.037 | 0.784、-0.781、0.811、-0.805、-0.840、-0.839、0.738 |
| Acinetobacter | M5 | cellulase、Glucosidase、α-amylase | | 0.000、0.000、0.002 | 0.989、0.948、0.904 |
| Thauera | M8 | cellulase、Glucosidase、α-amylase | | 0.000、0.000、0.002 | 0.984、0.960、0.906 |
| Methylobacter | M10 | | acidity、cellulase、Glucosidase、α-amylase | 0.048、0.001、0.001、0.002 | -0.712、0.936、0.928、0.909 |
| SBR1031 | M12 | cellulase、Glucosidase、α-amylase | | 0.003、0.006、0.003 | 0.890、0.858、0.888 |
| Macellibacteroides | M14 | cellulase、Glucosidase、α-amylase | | 0.000、0.001、0.001 | 0.980、0.930、0.918 |
| A4b | M18 | cellulase、Glucosidase、α-amylase | | 0.006、0.019、0.011 | 0.862、0.793、0.828 |
| Bacteroidetes_vadinHA17 | M19 | α-amylase | | 0.021 | 0.785 |
| Aspergillus | M23 | starch、reducing sugar、acidity、acid protease | | 0.012、0.028、0.017、0.012 | -0.825、-0.762、0.801、0.822 |
| Pseudallescheria | M33 | cellulase、Glucosidase、α-amylase | | 0.000、0.001、0.003 | 0.995、0.924、0.887 |
| control group | | | | | |
| Lysinibacillus | N3 | Moisture、starch、reducing sugar、acidity、cellulase、Glucosidase、α-amylase | | 0.021、0.013、0.012、0.007、0.012、0.022、0.000 | -0.786、0.818、0.824、-0.854、0.821、0.780、0.972 |
| Kocuria | N16 | acid protease | | 0.028 | 0.763 |
| Paracoccus | N17 | acid protease | | 0.028 | 0.761 |
| Kazachstania | N21 | Moisture、starch、reducing sugar、acidity、cellulase、Glucosidase、α-amylase、acid protease | | 0.008、0.009、0.043、0.013、0.012、0.042、0.037、0.035 | -0.849、0.843、0.722、-0.821、0.823、0.725、0.738、-0.742 |
| Knufia | N25 | acid protease | | 0.042 | 0.726 |
| Nothophoma | N27 | acid protease | | 0.03 | 0.757 |
| Neocucurbitaria | N30 | acid protease | | 0.041 | 0.726 |
| Cephalotrichum | N35 | reducing sugar | | 0.019 | 0.793 |
| Alternaria | N38 | acid protease | | 0.039 | 0.732 |
| Saccharomyces | N39 | Moisture、starch、Glucosidase | | 0.03、0.036、0.024 | -0.755、0.738、0.774 |
